# Stress-Enhanced Fear Learning in Rodents: A Systematic Review and Meta-Analysis of Fear-Learning Sensitization After Severe Stress

**DOI:** 10.3390/brainsci16070691

**Published:** 2026-06-30

**Authors:** Ruiying Liu, Gengxian He, Jing Pan, Shanni Fu, Shuairong Lin, Xinying Zhang, Shou Qiu, Zhijie Zhu, Xiaoyan Zhu, Jialu Feng

**Affiliations:** School of Medicine, Wuhan University of Science and Technology, Wuhan 430065, China; liuruiying@wust.edu.cn (R.L.);

**Keywords:** stress-enhanced fear learning, posttraumatic stress disorder, trauma-enhanced fear learning, fear conditioning, fear sensitization, freezing behavior, rodent model, meta-analysis, protocol sensitivity, translational interpretation

## Abstract

**Highlights:**

**What are the main findings?**

- Across 25 studies and 49 comparisons, prior severe stress was associated with higher later freezing in rodents.
- Protocol- and sample-level variables, including species, strain, stress method, testing interval, and stress type, contributed to variation in the pooled effect.

**What are the implications of the main findings?**

- SEFL is most useful as a focused paradigm of trauma-enhanced subsequent fear learning rather than a full PTSD model.
- Greater protocol standardization and stronger intervention evidence are needed before predictive validity claims can be strengthened.

**Abstract:**

**Background/Objectives**: Stress-enhanced fear learning (SEFL) tests whether severe stress amplifies a later, separate fear-learning episode. Although SEFL studies commonly report increased freezing, its overall effect size, robustness, protocol-level variation, and translational interpretation have not been systematically quantified. This review evaluated SEFL as a focused paradigm of stress-induced fear-learning sensitization rather than as a comprehensive model of posttraumatic stress disorder (PTSD). **Methods**: Web of Science Core Collection, PubMed, Embase, and Scopus were searched from inception to June 2026. Eligible studies used an SEFL-related procedure, included independent stress-exposed and control groups, and reported extractable freezing outcomes during later fear testing. Hedges’ g values were pooled using restricted maximum-likelihood random-effects models. We also examined exploratory subgroup analyses, sensitivity analyses, and small-study-effect assessments. **Results**: Twenty-five studies comprising 49 comparisons were included. Stress-exposed animals showed higher freezing than controls during later fear testing (Hedges’ g = 1.71, 95% CI [1.38, 2.04], *p* < 0.001). Heterogeneity was substantial (I^2^ = 81.91%, τ^2^ = 1.07), and the 95% prediction interval crossed zero (−0.40 to 3.82), indicating that SEFL is not protocol-invariant. Exploratory subgroup analyses suggested protocol- and sample-level variation, particularly by species, strain, stress method, testing interval, and stress type. Contextual-fear-conditioning-only, study-level, non-footshock-exclusion, and leave-one-out sensitivity analyses supported the overall positive effect, although possible small-study effects should be considered when interpreting the pooled estimate. **Conclusions**: SEFL is best interpreted as a protocol-sensitive behavioral neuroscience paradigm for studying stress-enhanced later fear learning after severe stress rather than as a broad PTSD model. The current evidence supports a large average freezing-defined SEFL effect, but the marked heterogeneity and limited intervention-related evidence indicate that the paradigm should be interpreted with attention to protocol conditions and translational scope.

## 1. Introduction

### 1.1. Process Boundaries of PTSD and Limitations of Animal Models

Posttraumatic stress disorder (PTSD) is related to traumatic stress exposure, but it is not defined by stress exposure alone. In DSM-5 and DSM-5-TR, PTSD is classified as a trauma- and stressor-related disorder that may follow direct or indirect exposure to traumatic events, including actual or threatened death, serious injury, or sexual violence [1,2]. Its clinical presentation includes intrusion, avoidance, negative alterations in cognition and mood, and alterations in arousal and reactivity [1,2]. For a diagnosis, symptoms must persist for more than one month and cause clinically significant distress or functional impairment [1,2]. Fear learning, extinction, extinction recall, safety learning, and fear generalization are widely studied processes relevant to fear-, anxiety-, trauma-, and stressor-related disorders [3,4,5,6,7]. These fear- and threat-related processes are also transdiagnostic and align with dimensional frameworks such as the Research Domain Criteria (RDoC), rather than mapping uniquely onto DSM-defined PTSD [8,9].

Rodent models cannot reproduce PTSD in full. Nightmares, intrusive memories, guilt, and distorted self-beliefs depend on subjective report and cannot be measured directly in animals. Animal studies therefore rely on behavioral readouts such as freezing, startle, social interaction, exploration, and responses to trauma-associated contexts [10,11,12]. Other tasks, including sucrose preference, forced-swim immobility, and Morris water maze, can provide information about stress-related affective or cognitive changes [12,13,14,15,16]. These outcomes are useful, but they represent selected fear-, threat-, affective, or cognitive processes rather than PTSD as a whole.

The stressor itself also affects how an animal model should be interpreted. A short, predictable, or rather mild exposure is likely to not give rise to the same phenotype as a long, strong, uncontrollable, or unpredictable one. It is particularly important in footshock paradigms that the experimenter specifies shock number, intensity, and timing. If the procedure is weak, later freezing reflects transient reactivity to stress rather than a more permanent change in fear learning. If it is too strong or poorly calibrated, injury, ceiling effects, or nonspecific behavioral disruption may complicate the result. Similar worries are raised in the context of other stress-based paradigms, like restraint and predator-stress procedures, which have effects that can differ significantly by exposure method and physiological challenge [17,18].

More broadly, the value of an animal model depends less on whether it resembles PTSD in full than on whether it isolates the process being studied. For trauma-related enhancement of fear learning, the relevant paradigm should separate fear of the original stressor from increased vulnerability to later aversive learning. Learned helplessness and rodent early adversity paradigms illustrate related but different questions: controllability in one case and developmental vulnerability in the other [19,20,21,22,23,24]. These lines of work are useful, but they are less direct than SEFL for isolating the effect of prior trauma-like stress on later fear learning. Stress-enhanced fear learning (SEFL) is therefore relevant to this review because it isolates this specific PTSD-relevant process: whether a prior trauma-like stressor increases vulnerability to later fear learning.

### 1.2. Why SEFL Requires a Focused Synthesis

SEFL addresses this specific problem by examining how a severe stress experience alters later fear learning [25,26,27]. In a typical design, animals first receive severe stress in context A and are later given a milder conditioning event in a distinct context B [25,26]. This separation preserves the experimental control of Pavlovian fear conditioning while shifting the focus away from normal fear acquisition and toward the longer-lasting consequences of prior stress. If animals freeze more in context B after prior stress exposure, the result does not necessarily imply fear of the original shock context. Instead, it suggests that the previous stress experience has made the animal more susceptible to later aversive learning.

Rajbhandari et al. [27] describe SEFL as a three-phase procedure: severe stress in context A, mild fear conditioning in a distinct context B, and later fear testing in context B. Freezing in the final test is usually considered the main behavioral readout. This design directly asks whether prior severe stress enhances a later fear-learning episode. The effect can persist well beyond the original stress exposure, with some studies reporting SEFL-related enhancement for up to 90 days [26]. A commonly used rat protocol involves 15 footshocks in context A during the trauma-like session, followed by one shock in context B [27]. Rau et al. [25] reported that animals receiving 15 shocks froze more during later testing than animals receiving a brief single shock, three massed shocks, or 15 spaced shocks. In SEFL, the first shock session is not assessed as conventional fear conditioning in itself; instead, it is used to alter the animal’s response to the later, milder conditioning episode.

One commonly used SEFL protocol involves relatively intense shock exposure, and later work has refined the procedure by modifying shock number and shock intensity [28]. These refinements are relevant to both animal welfare and model interpretation: they help define the model’s limits while remaining consistent with general refinement principles [29,30]. Depending on how the stressor is calibrated, the same SEFL framework may produce durable fear-learning sensitization, a short-lived stress response, or nonspecific behavioral disruption.

SEFL also raises the question of individual and procedural differences. Some animals may show a stronger SEFL-like phenotype than others, and susceptibility may vary with parameters of stress, strain, sex-related factors, and the behavioral endpoint [28,31,32]. Individual experiments can show that SEFL occurs under specific conditions. However, they cannot show how consistent the phenotype is across laboratories or which protocol factors most strongly shape its effect size.

Therefore, a focused synthesis of the SEFL literature is needed. Several narrative reviews have discussed SEFL and closely related stress-enhanced fear-learning paradigms. Nishimura et al. [11] describe SEFL as a useful approach for distinguishing later fear-learning sensitization from fear memory for the original stressor. Blouin et al. review possible epigenetic mechanisms relevant to stress, fear memory, and SEFL-related PTSD models [33]. Broader reviews also highlight how stress, sex, and fear learning interact [34,35]. These papers provide important background for interpreting SEFL, but they do not pool freezing outcomes across SEFL studies or estimate how much the effect varies across protocols. We therefore complement this literature by quantitatively summarizing the average SEFL effect and its cross-study variability. SEFL’s main strength is its specificity: it targets the long-lasting enhancement of later fear learning after prior severe stress [25,26,27]. However, its cross-laboratory stability and the protocol factors shaping effect size remain unclear, because SEFL protocols differ in shock number, shock intensity, conditioning modality, testing interval, strain, and sex. Claims about translational interpretation and model-relevant features of SEFL have been made in individual studies, but these claims have rarely been organized across studies. This review uses meta-analysis to estimate the overall SEFL effect and to examine the main sources of variation. Other PTSD-related animal paradigms are discussed only insofar as they help clarify the scope of SEFL and interpret the quantitative findings.

### 1.3. Model-Relevant Framework and Study Aims

A rodent paradigm is most informative when it is matched to the question being asked. It does not need to reproduce posttraumatic stress disorder (PTSD) as a whole, but it should isolate a process that can be measured and interpreted in animals. Classical animal-model terminology, including face, construct, and predictive validity, can be useful for discussing the translational scope of a paradigm, but these terms were used here as orientation points rather than as formal scoring criteria or as a checklist for an ideal PTSD model [36,37,38]. In this terminology, face validity concerns observable phenotype resemblance, construct validity concerns the modeled causal or biological process, and predictive validity concerns whether a model responds to clinically relevant interventions [36,38]. For SEFL, these terms require especially cautious use because the paradigm primarily measures freezing after later fear testing and does not capture the full clinical phenotype of PTSD.

Accordingly, this review did not aim to declare SEFL a valid model of PTSD. This review focuses on fear acquisition and sensitization, that is, the enhancement of later fear learning after prior severe stress. Extinction is highly relevant to PTSD and to the persistence or recovery of conditioned fear [7,39], but extinction outcomes were not consistently reported in the eligible SEFL studies and were therefore not treated as a primary endpoint in the present analysis. Instead, we quantified the overall effect of SEFL on later freezing behavior and examined exploratory protocol-level sources of variation across studies. We also summarized model-relevant features of the literature, including the freezing phenotype, the context-separated procedural design, and intervention-related evidence. This scope guided our systematic review and meta-analysis of rodent SEFL studies and was used to clarify what the current evidence can and cannot support regarding SEFL as a focused paradigm of stress-enhanced later fear learning.

## 2. Materials and Methods

### 2.1. Data and Software

We performed the meta-analyses using Stata 18.0 (StataCorp LLC, College Station, TX, USA) and used Review Manager 5.4 (The Cochrane Collaboration, London, UK) to prepare the risk-of-bias figures.

### 2.2. Review Protocol and Reporting

We conducted and reported the meta-analysis according to the Preferred Reporting Items for Systematic Reviews and Meta-Analyses (PRISMA) 2020 statement [40], and the completed PRISMA 2020 checklist is provided as Supplementary File S1. The protocol was registered in PROSPERO (CRD420251075443). In this article, narrative content is used to clarify the scope of SEFL as a rodent paradigm of stress-induced enhancement of later fear learning, while the quantitative synthesis is limited to SEFL studies with extractable freezing outcomes. The main quantitative eligibility criteria were not broadened after protocol registration. During revision, we expanded the search strategy to improve sensitivity for SEFL-specific studies and added sensitivity analyses in response to reviewer concerns regarding comparison-level dependence, conditioning modality and the influence of non-footshock stressor comparisons.

### 2.3. Literature Search

We searched four databases: PubMed, Web of Science Core Collection, Embase, and Scopus. Only English-language articles were included. The original search covered the period from database inception to June 2026, with PubMed and Web of Science Core Collection searched to 16 June 2026 and Embase and Scopus searched to 18 June 2026. During revision, we reran the original search strategy and conducted an expanded SEFL-specific search to improve sensitivity for studies that used stress-enhanced fear learning procedures but did not necessarily frame the work using PTSD-related terminology. The expanded search did not broaden the quantitative eligibility criteria; it was used to identify additional eligible rodent SEFL studies that may have been missed by a search strategy requiring trauma- or PTSD-related terms. Reference-list checking did not identify additional eligible records beyond those captured in the database searches. The complete original and expanded search strategies are reported in the Supplementary Materials.

### 2.4. Eligibility Criteria, Study Selection, and Comparison Definition

Eligible studies had to use a SEFL-related procedure, include independent stress-exposed and control groups, assess later fear conditioning and testing after prior stress exposure, and report quantifiable freezing outcomes during fear testing. Studies involving pharmacological, genetic, surgical, optogenetic, chemogenetic, or other manipulations were excluded unless a standard SEFL comparison using non-genetically modified animals and without the additional manipulation could be extracted. Reviews, commentaries, meeting abstracts, and studies without sufficient data for effect-size calculation were excluded. An exception was made only when a review-type article reported original, extractable animal experimental data that otherwise met all quantitative eligibility criteria. In such cases, only the original experimental comparison was extracted; narrative summaries of previously published studies were not treated as independent data. Two reviewers independently conducted study screening, and disagreements were resolved through discussion with a third reviewer. When more than one eligible comparison was obtained from the same parent study, the comparisons retained the same parent study ID but were assigned unique comparison IDs. The eligible comparisons considered for selection were defined as one per stress-exposed group versus one control group from the same parent study, species, strain, sex category, stress method, conditioning, and testing interval. Detailed comparison IDs, parent study labels, reference information, and shared-control adjustments are provided in Supplementary Table S2.

Eligible animals were non-genetically modified rats or mice of either sex and any age. Human studies, in vitro, in silico, and ex vivo studies were excluded, as were studies using animal species other than rats or mice. Eligible control groups included rats or mice that underwent the same conditioning and testing procedures as the stress-exposed animals but did not receive prior stress exposure.

### 2.5. Data Extraction and Model-Relevant Feature Coding

For each eligible comparison, we extracted the sample size, animal species and strain, sex, age or developmental stage at stress exposure, conditioning, and/or fear testing when reported, stress protocol, stress type, conditioning type, testing interval, and the mean and standard deviation of freezing behavior in the stress-exposed and control groups. Age and developmental-stage information was summarized descriptively because reporting was inconsistent and age was often confounded with other protocol features. When numerical values were not provided in the text or tables, data were extracted from graphs using WebPlotDigitizer, version 4.7 (Ankit Rohatgi, Pacifica, CA, USA) [41]. When standard errors of the mean were reported, standard deviations were calculated by multiplying the standard error by the square root of the corresponding group sample size. The primary effect direction was defined as the stress-exposed group minus the control group; therefore, positive Hedges’ g values indicate higher freezing in stress-exposed animals than in controls. Data extraction was performed independently by two reviewers, and any discrepancies were resolved through discussion with a third reviewer.

When a study reported more than one eligible comparison or outcome, we applied predefined extraction rules. First, if freezing was reported at several time points within a test session, the value closest to the start of the session was used. This rule was used to capture the initial expression of conditioned freezing before within-session extinction, habituation, or repeated testing could alter freezing levels. Second, if fear testing was conducted more than once, data from the first test were extracted. Third, if the sample size was reported as a range, the minimum value was used. Fourth, when multiple intervention conditions were included, the SEFL group without additional manipulations was prioritized. Finally, when several stress-exposed groups were available, the authors’ primary SEFL condition was selected when identifiable; otherwise, the group most consistent with the predefined extraction criteria and commonly used SEFL procedure was extracted. Modified footshock procedures were coded when footshock-based stress procedures differed from the commonly used 15-shock SEFL procedure in one or more features, including shock number, shock intensity, shock duration, or session structure. Non-footshock stress procedures were coded separately.

Alongside standard data extraction, we coded descriptive model-relevant features to organize evidence relevant to the translational interpretation of SEFL. For observable fear- or threat-response phenotype, we recorded increased freezing, persistence of the effect, and any additional behaviors related to threat exaggeration, avoidance, or reduced exploration. For core procedural logic, we recorded whether severe stress exposure and later conditioning or testing occurred in separated contexts, whether the procedure followed the core three-stage logic of SEFL, and whether the effect varied with protocol-level variables. For intervention-related evidence, we recorded intervention type, timing, outcome direction, and relevance to translational interpretation. Intervention-related evidence was summarized qualitatively and was not pooled in the main meta-analysis. Feature coding was checked against the extracted study characteristics and original study aims. The full study-level model-relevant feature coding is provided in Supplementary Table S3. These descriptive codes were used to organize model-relevant evidence rather than to generate a separate quantitative validity score, and they were not treated as formal evidence that SEFL fulfills classical construct or predictive validity criteria.

### 2.6. Data Synthesis and Statistical Analysis

We used the comparison, rather than the study, as the unit of analysis. Each eligible contrast between stress-exposed and control animals contributed one effect size. Hedges’ g was calculated from the mean, standard deviation, and sample size of each group to reduce small-sample bias in standardized mean differences [42]. The stress-exposed group was entered first so that positive values indicated higher freezing in stress-exposed animals than in controls. Because SEFL studies differed in strain, stress protocol, conditioning procedure, and testing conditions, random-effects models with 95% confidence intervals (CI) were used [42]. The restricted maximum likelihood (REML) estimator was used as the primary method for estimating between-comparison variance [43].

When a single control group was shared by multiple stress-exposed groups, the sample size of the control group was divided by the number of relevant comparisons to avoid double-counting animals, in accordance with recommended approaches for handling multiple intervention groups in meta-analysis [44,45]. These adjusted control sample sizes were used for effect-size calculation in the primary meta-analysis. To examine potential sources of between-comparison variability, exploratory subgroup analyses were performed according to species group, strain, sex, conditioning type, stress method, stress type, and testing interval. Because several strain subgroups contained few comparisons, strain-level analyses were considered exploratory. Subgroup analyses were used to describe possible sources of heterogeneity and were not interpreted as definitive tests of biological species, strain, sex, stress-method, or conditioning-modality differences.

Forest plots were used to display individual and pooled effect sizes. Heterogeneity was assessed using Cochran’s Q test, I^2^, τ^2^, and H^2^ [42,46]. We also calculated 95% prediction intervals to estimate the range of effects expected across future comparable SEFL protocols [47]. Between-subgroup differences in subgroup analyses were assessed using the Qb statistic. Sensitivity analyses included leave-one-out analysis, a contextual fear conditioning (CFC)-only sensitivity analysis, and a study-level sensitivity analysis retaining one representative comparison per study. An additional exploratory sensitivity analysis excluded non-footshock stressor comparisons. For the study-level sensitivity analysis, we prioritized the primary SEFL comparison reported by the authors. When several eligible comparisons were available, we selected the comparison most closely matching the core SEFL design, including independent stress-exposed and control groups, vehicle or no-intervention conditions, contextual fear testing where available, and the earliest post-conditioning test used to assess SEFL expression. Because contextual and auditory fear conditioning differ in their behavioral and neural features and because auditory fear-conditioning comparisons were few, we conducted a CFC-only sensitivity analysis. Small-study effects were assessed using funnel plots and Egger’s regression test [48]. Trim-and-fill analysis was conducted to evaluate the potential influence of missing studies on the pooled estimate [49]. All analyses were conducted using Stata 18.0 (StataCorp LLC, College Station, TX, USA).

### 2.7. Assessment of Risk of Bias

We assessed the risk of bias in the included studies using the risk-of-bias tool developed by the Systematic Review Centre for Laboratory Animal Experimentation (SYRCLE) [50]. This tool is adapted from the Cochrane Risk of Bias tool and evaluates domains such as randomization, allocation concealment, blinding, incomplete outcome data, selective reporting, and other sources of bias. Each domain was judged as low, high, or unclear risk. Two reviewers independently assessed risk of bias, and disagreements were resolved by discussion or consultation with a third reviewer. We did not contact study authors for additional methodological information; therefore, items that were not reported in the published article or Supplementary Materials were coded as unclear rather than absent.

## 3. Results

Twenty-five studies met the eligibility criteria for quantitative synthesis, contributing 49 eligible comparisons. We first describe study selection, study and comparison characteristics, and risk of bias, and then report the overall pooled effect, exploratory subgroup analyses, sensitivity analyses, and small-study-effect assessments.

### 3.1. Study Selection

**Figure 1:**
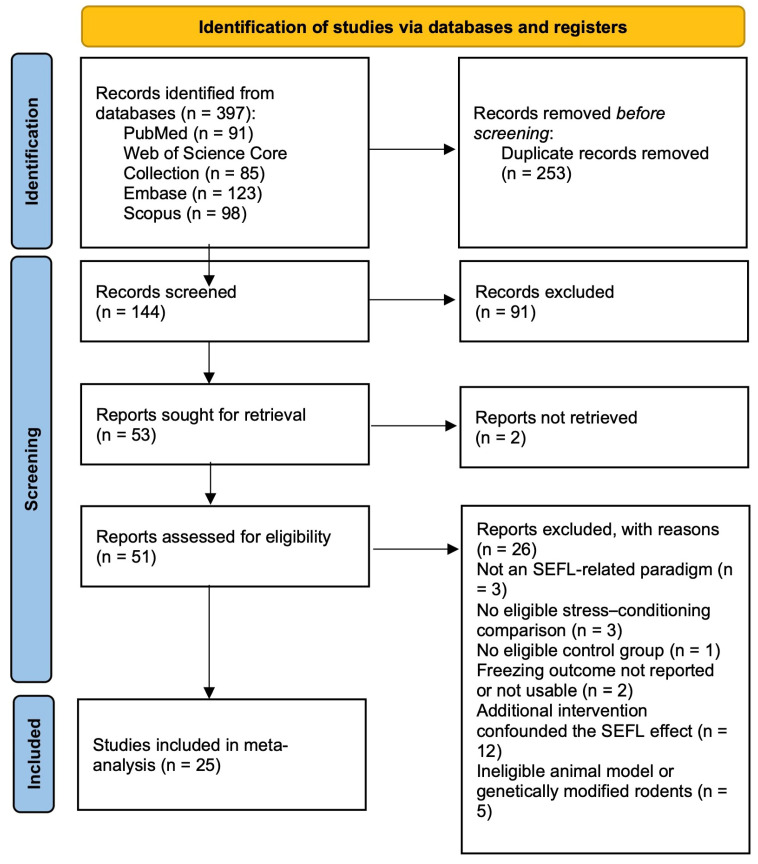
PRISMA 2020 flow diagram showing identification, deduplication, screening, full-text assessment, exclusion reasons, and inclusion of SEFL studies in the revised meta-analysis.

A total of 397 records were identified from four databases: PubMed (*n* = 91), Web of Science Core Collection (*n* = 85), Embase (*n* = 123), and Scopus (*n* = 98). After 253 duplicate records were removed, 144 records were screened by title and abstract, and 91 records were excluded. Fifty-three reports were sought for retrieval, of which two could not be obtained. The remaining 51 full-text reports were assessed for eligibility. Twenty-six reports were excluded with reasons: not an SEFL-related paradigm (*n* = 3), no eligible stress–conditioning comparison (*n* = 3), no eligible control group (*n* = 1), freezing outcome not reported or not usable (*n* = 2), additional intervention confounded the SEFL effect (*n* = 12), and ineligible animal model or genetically modified rodents (*n* = 5). Overall, 25 studies were included in the meta-analysis. These studies contributed 49 eligible comparisons. The study selection process is summarized in Figure 1.

### 3.2. Study and Comparison Characteristics

The 25 included studies comprised 49 comparisons and were published between 2005 and 2025 [10,25,32,51,52,53,54,55,56,57,58,59,60,61,62,63,64,65,66,67,68,69,70,71,72]. At the comparison level, the studies varied in animal species and strain, sex, conditioning type, stress method, stress type, and the interval between stress exposure and fear testing. Rats accounted for most comparisons (*n* = 44), whereas mice accounted for five comparisons. Contextual fear conditioning (CFC) was used in most comparisons (*n* = 46), whereas auditory fear conditioning (AFC) was used in three comparisons. Acute stress was used in most comparisons (*n* = 47), while chronic stress was used in two comparisons. Classic footshock was the most frequently used stress method (*n* = 29), followed by modified footshock protocols (*n* = 9), non-footshock stressors (*n* = 9), and chronic footshock exposure (*n* = 2).

With respect to sex, 29 comparisons included male animals, eight included female animals, and 12 included both sexes. The timing of fear testing also varied: 20 comparisons were tested within 3 days after stress exposure, five were tested between 4 and 7 days, 10 between 8 and 14 days, five between 15 and 30 days, and nine after more than 30 days. Descriptive model-relevant feature coding is provided in Supplementary Table S3.

Table 1 summarizes the main characteristics of the included comparisons. Detailed comparison-level extraction data, including comparison IDs, parent study labels, and shared-control adjustments, are provided in Supplementary Table S2.

### 3.3. Risk of Bias

Risk of bias was assessed for the 25 included studies using SYRCLE’s risk-of-bias tool. Several domains showed relatively low risk of bias. Baseline characteristics and selective outcome reporting were judged as low risk in all included studies (100%, 25/25). Incomplete outcome data were judged as low risk in 100.0% of studies (25/25). Blinding of outcome assessment was judged as low risk in 80.0% of studies (20/25) and unclear risk in 20.0% of studies (5/25). Random sequence generation was judged as low risk in 36.0% of studies (9/25), whereas the remaining studies were rated as unclear because randomization procedures were not sufficiently described.

Unclear risk of bias was common in several methodological domains. Allocation concealment, random housing, blinding of caregivers/investigators, and random outcome assessment were rated as unclear in all included studies (100%, 25/25), mainly because these details were not reported. Other bias was judged as low risk in 8.0% of studies (2/25) and unclear risk in 92.0% of studies (23/25). The high proportion of unclear ratings across key domains, including allocation concealment, random housing, and blinding of caregivers or investigators, reflects insufficient reporting and limits confidence in the internal validity of the included studies. The risk-of-bias assessment is provided in Supplementary Figure S1, and detailed study-level judgments are provided in Supplementary Table S4.

### 3.4. Overall SEFL Effect on Freezing Behavior

**Figure 2:**
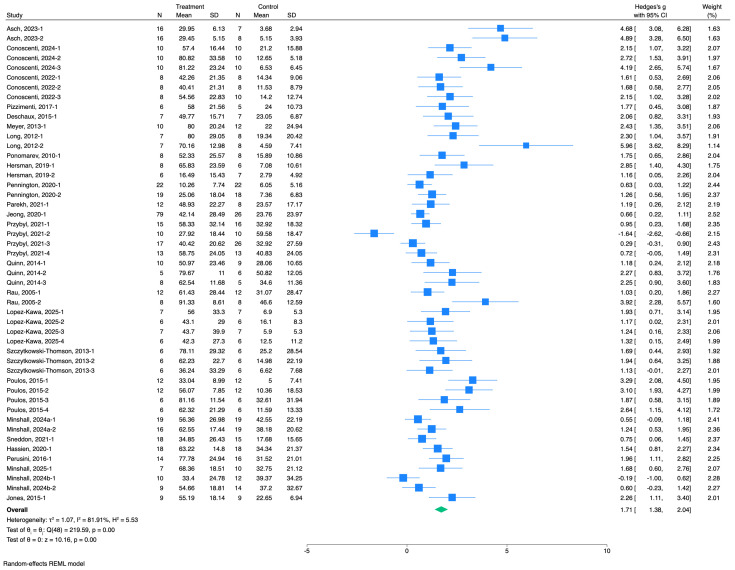
Forest plot of the overall SEFL effect using a REML random-effects model. Effect sizes are shown as Hedges’ g with 95% confidence intervals. The pooled estimate was Hedges’ g = 1.71, 95% CI [1.38, 2.04]. Blue squares represent the effect estimates for individual comparisons, with the square size reflecting the relative weight. The green rhombus represents the pooled effect estimate. The author–year labels shown in the forest plot correspond to the following references: Hassien et al. [10], Rau et al. [25], Lopez-Kawa and Lattal [32], Szczytkowski-Thomson et al. [51], Long and Fanselow [52], Minshall et al. 2024a [53], Poulos et al. [54], Asch et al. [55], Conoscenti et al. 2022 [56], Conoscenti et al. 2024 [57], Pizzimenti et al. [58], Deschaux et al. [59], Meyer et al. [60], Ponomarev et al. [61], Hersman et al. [62], Pennington et al. [63], Parekh et al. [64], Jeong et al. [65], Przybyl et al. [66], Quinn et al. [67], Sneddon et al. [68], Perusini et al. [69], Minshall et al. 2025 [70], Minshall et al. 2024b [71], and Jones et al. [72].

Forty-nine comparisons contributed to the overall analysis. Under the REML random-effects model, stress-exposed animals showed higher freezing than control animals (Hedges’ g = 1.71, 95% CI [1.38, 2.04], z = 10.16, *p* < 0.001). Heterogeneity was substantial (I^2^ = 81.91%, τ^2^ = 1.07; H^2^ = 5.53; Cochran’s Q(48) = 219.59, *p* < 0.001). The 95% prediction interval ranged from −0.40 to 3.82 and crossed zero, indicating that SEFL may be absent or markedly reduced under some experimental settings despite the large average effect. Although the average direction of effect was clearly positive, the magnitude of the effect varied substantially across study conditions. The overall REML forest plot is shown in Figure 2.

### 3.5. Exploratory Subgroup Analyses

Exploratory subgroup analyses suggested variation across several protocol- and sample-level variables, including species, strain, stress method, testing interval, and stress type. Stress types and species groups also varied across subgroups, although these results are difficult to interpret firmly because some categories were small or confounded with study-level design features. The subgroup tests for sex and conditioning type were not statistically significant, but this should not be read as evidence that sex-related influences are absent or that AFC and CFC outcomes are equivalent. Table 2 summarizes the exploratory subgroup results.

#### 3.5.1. Species and Strain

Species and strain were studied descriptively as potential sources of heterogeneity. The pooled estimate was larger in rat comparisons than in mouse comparisons. This pattern should be treated as preliminary because only five comparisons were included in the mouse subgroup, and species was confounded with protocol, strain, laboratory, and study-level design features. Strain-level estimates also varied across categories, but several strain subgroups contained few comparisons; therefore, these results should be treated as exploratory rather than confirmatory.

#### 3.5.2. Grouped Stress Method

Stress method showed exploratory between-subgroup variation. Classic, chronic, and modified footshock procedures showed positive pooled estimates, whereas non-footshock stressors showed a smaller but still significant pooled effect. This pattern suggests that stress method is one source of protocol-level variation in SEFL expression. The chronic-footshock subgroup should be interpreted especially cautiously because it was based on only two comparisons. Similarly, the smaller estimate observed for non-footshock stressors should be interpreted as a possible boundary-condition signal rather than definitive evidence against non-footshock SEFL-like effects, because this subgroup was small and procedurally heterogeneous.

#### 3.5.3. Sex

The sex subgroup test was not statistically significant (Qb = 0.27, *p* = 0.876). Positive pooled estimates were observed in male-only comparisons (k = 29, Hedges’ g = 1.71, 95% CI [1.33, 2.09]), female-only comparisons (k = 8, Hedges’ g = 1.49, 95% CI [0.10, 2.88]), and comparisons including both sexes (k = 12, Hedges’ g = 1.83, 95% CI [1.31, 2.34]). These findings do not rule out sex-related influences, because sex-specific designs, estrous-cycle information, and susceptibility profiles were not consistently available across studies [31,32].

#### 3.5.4. Conditioning Type

The conditioning-type subgroup test was not statistically significant in this dataset (Qb = 1.30, *p* = 0.255). Positive pooled effects were observed in both AFC comparisons (k = 3, Hedges’ g = 1.21, 95% CI [0.34, 2.07]) and CFC comparisons (k = 46, Hedges’ g = 1.75, 95% CI [1.40, 2.10]). However, the AFC subgroup included only three comparisons. The current data therefore do not support a definitive conditioning-specific difference in SEFL expression, and the absence of a statistically significant subgroup effect should not be interpreted as evidence that AFC and CFC outcomes are equivalent.

#### 3.5.5. Testing Interval

Testing interval showed exploratory between-subgroup variation (Qb = 19.41, *p* = 0.001). Positive pooled effects were observed across all interval categories, but their magnitude varied over time rather than following a simple linear pattern. The largest pooled estimate appeared at 4–7 days (k = 5, Hedges’ g = 2.86, 95% CI [2.02, 3.70]), followed by 15–30 days (k = 5, Hedges’ g = 2.35, 95% CI [1.10, 3.60]). Effects beyond 30 days were smaller but remained significant (k = 9, Hedges’ g = 0.93, 95% CI [0.51, 1.35]). These findings suggest that SEFL can persist, although the measured strength of the phenotype depends on the timing of fear testing.

#### 3.5.6. Stress Type

Stress type showed exploratory between-subgroup variation (Qb = 5.29, *p* = 0.022). Acute stress comparisons showed a significant pooled effect (k = 47, Hedges’ g = 1.64, 95% CI [1.32, 1.97]), whereas chronic stress comparisons showed a larger estimate (k = 2, Hedges’ g = 3.37, 95% CI [1.93, 4.81]). This result should be read as exploratory because the chronic-stress subgroup included only two comparisons from the same study and overlapped with the chronic-footshock subgroup. It is therefore insufficient to separate chronic stress exposure from the specific chronic-footshock procedure.

### 3.6. Sensitivity Analyses

The main estimate was supported by sensitivity analyses. A CFC-only sensitivity analysis retained a significant pooled effect across 46 comparisons (Hedges’ g = 1.75, 95% CI [1.40, 2.10], z = 9.80, *p* < 0.001). Heterogeneity remained substantial (τ^2^ = 1.14, I^2^ = 81.90%; Q(45) = 209.03, *p* < 0.001). This analysis indicates that the main finding was not driven solely by combining contextual and auditory/cued fear-conditioning outcomes.

A study-level sensitivity analysis retaining one representative comparison per study also yielded a significant positive effect across 25 studies (Hedges’ g = 1.58, 95% CI [1.22, 1.94], z = 8.54, *p* < 0.001). Heterogeneity was reduced but remained substantial (τ^2^ = 0.59, I^2^ = 74.23%; Q(24) = 85.65, *p* < 0.001). This analysis suggests that the overall positive SEFL effect was not solely driven by studies contributing multiple comparisons.

An additional exploratory sensitivity analysis excluding non-footshock stress comparisons also retained a significant positive effect across 40 comparisons (Hedges’ g = 1.92, 95% CI [1.57, 2.27], z = 10.89, *p* < 0.001). Heterogeneity remained substantial (τ^2^ = 0.90, I^2^ = 76.68%; Q(39) = 151.31, *p* < 0.001). This analysis indicates that the overall positive effect was not dependent on the inclusion of non-footshock stress comparisons.

The leave-one-out analysis did not identify any single influential comparison. When each of the comparisons was removed one at a time, the pooled Hedges’ g ranged from 1.647 to 1.759. In all cases, the 95% confidence intervals remained above zero, and all *p* values were <0.001. The overall positive effect therefore remained stable after accounting for conditioning modality, study-level dependence, non-footshock stressor inclusion, and single-comparison influence.

### 3.7. Small-Study Effects

Small-study effects were assessed using funnel plots, Egger’s regression test, and trim-and-fill analysis. Egger’s regression was significant (β = 5.60, SE = 0.694, z = 8.07, *p* < 0.001), suggesting possible small-study effects. Given the substantial heterogeneity, this result should be interpreted cautiously and should not be taken as direct evidence of publication bias. The pooled estimate was reduced after 17 comparisons were imputed to the left side of the funnel plot. The adjusted effect was still significant (Hedges’ g = 1.10; 95% CI [0.71, 1.49]). The results indicate that this pooled estimate may have been inflated by small-study effects, though those effects cannot fully account for the overall positive effect. The sensitivity analyses and small-study-effect assessments are summarized in Table 3.

## 4. Discussion

This meta-analysis examined how robust the SEFL effect is across rodent studies and which protocol conditions shape its magnitude. Across 25 studies and 49 comparisons, stress-exposed animals showed a clear increase in later freezing under the REML random-effects model (Hedges’ g = 1.71, 95% CI [1.38, 2.04], z = 10.16, *p* < 0.001; τ^2^ = 1.07; I^2^ = 81.91%; 95% prediction interval = −0.40 to 3.82). The effect was not uniform, however. The substantial heterogeneity and the 95% prediction interval, which crossed zero, indicate that SEFL should not be treated as a protocol-invariant effect and may be absent or markedly reduced under some experimental conditions. For this reason, the pooled estimate is best read as an average across different SEFL implementations, not as an effect expected under every protocol. The sensitivity analyses supported this interpretation. The CFC-only analysis retained a significant overall effect, indicating that the main finding was not driven solely by combining contextual and auditory/cued fear-conditioning outcomes. The study-level sensitivity analysis, which retained one representative comparison per study, also remained significant, suggesting that the overall effect was not solely driven by studies contributing multiple comparisons. The non-footshock-exclusion and leave-one-out analyses further supported the stability of the positive pooled effect. Nevertheless, heterogeneity remained substantial across the main and sensitivity analyses.

These findings suggest that SEFL is best interpreted as a protocol-sensitive behavioral neuroscience paradigm for studying trauma-related fear-learning sensitization. The discussion below therefore focuses on the scope and limits of this paradigm, rather than on evaluating SEFL as a complete animal model of PTSD.

### 4.1. Observable Fear- and Threat-Response Expression

The pooled increase in freezing supports an average increase in fear- or threat-response expression, which was the only endpoint consistently available for quantitative synthesis. The positive effect beyond 30 days suggests that SEFL expression is not restricted to a short-lived acute stress reactivity, although the smaller estimate in this subgroup indicates that the strength of the phenotype may depend on testing interval.

This evidence should not be read as broad symptom coverage. Freezing is a useful endpoint, but it cannot stand for the full clinical picture of PTSD, especially symptoms that depend on subjective experience, such as intrusive memories, nightmares, guilt, or distorted self-beliefs. The present meta-analysis also did not pool avoidance, startle, sleep, reward, social, or cognitive outcomes. Some SEFL-related studies have reported avoidance-like behavior or reduced exploration, but these outcomes were not common endpoints across studies [10,11]. The phenotype-level interpretation should therefore be limited mainly to exaggerated fear- and threat-response expression.

### 4.2. Model-Relevant Procedural Features and Boundary Conditions

The process targeted by SEFL is whether trauma-like severe stress enhances later fear learning in a separate context [25,26,27]. The paradigm does not simply ask whether footshock increases freezing. Its central question is whether severe stress in one context makes later fear learning stronger in a separate context. This distinction matters because contextual fear generalization may involve associative components and should not be treated as identical to the stress-related sensitization process targeted by SEFL [73]. The exploratory subgroup findings can therefore be interpreted in relation to this process: whether prior severe stress enhances later fear learning in a separate context. The core SEFL design separates severe stress exposure from later mild conditioning and testing, often across distinct contexts.

Stress-related protocol variables also appeared to contribute to SEFL heterogeneity. The exploratory subgroup findings suggested that stress method may act as a source of variation, although this pattern should not be interpreted as evidence that one stress procedure is intrinsically superior. Variation across stress procedures may reflect stressor intensity, controllability, physiological challenge, species or strain composition, and other protocol-level design choices [74]. The sensitivity analysis excluding non-footshock stress comparisons further showed that the overall effect remained significant and became larger, indicating that the main finding was not dependent on non-footshock stressor comparisons. At the same time, the positive pooled estimate in the non-footshock subgroup suggests that these procedures should not be dismissed as invalid, but rather interpreted as procedurally heterogeneous and potentially weaker implementations of stress-enhanced later fear learning. The stress-type finding should also be interpreted cautiously because the chronic-stress subgroup was based on only two comparisons from the same study. Thus, the stress-type result is best viewed as a preliminary signal rather than as proof that chronic stress produces stronger SEFL than acute stress.

Testing interval also contributed to variation in SEFL effect size. Positive pooled effects were observed across interval categories, but the estimates did not follow a simple linear trend. The largest pooled estimate appeared at 4–7 days, followed by 15–30 days, whereas effects beyond 30 days were smaller but still significant. This pattern does not define a single optimal testing window, but it suggests that SEFL expression has a time-dependent structure. The phenotype can persist, but its measured strength depends on when fear testing is conducted.

Overall, the context-separated design of SEFL and the subgroup patterns fit the procedural logic of a paradigm designed to test stress-enhanced later fear learning. The study’s findings do not show classical construct validity, but they do suggest when SEFL will be most useful; only if the stress procedure, conditioning design, and testing interval are specified.

### 4.3. SEFL in Relation to Prior Literature

The pooled analysis showed a large average SEFL effect (Hedges’ g = 1.71), with stress-exposed animals freezing more during later fear testing than controls. This pattern is consistent with the core SEFL framework described in the SEFL literature [25,26,27]. At the same time, heterogeneity was high (I^2^ = 81.91%), and the 95% prediction interval crossed zero (−0.40 to 3.82). The effect is therefore strong on average, but not uniform across experimental conditions. This fits with prior work showing that SEFL can depend on stressor type, shock intensity, the interval between stress and conditioning, and the type of conditioning test used [26,28,74]. It also supports a cautious interpretation of SEFL as a protocol-sensitive paradigm for stress-enhanced later fear learning, rather than as a protocol-invariant PTSD model [11,28].

It is also important to separate SEFL from fear generalization in the narrower conditioning sense. Fear generalization usually refers to fear responses to cues or contexts that resemble the original conditioned stimulus or trauma-related setting [6,73]. SEFL asks a related but different question: whether severe stress in one context changes how strongly an animal learns a new aversive association in another context [25,26,27]. SEFL is therefore a form of stress-induced sensitization that may contribute to generalization-like phenomena, but it is not itself a measure of generalization to the original trauma context. Because the stress context and the later conditioning context are separated, increased freezing in the later test should not be read only as memory for the original stressor. It is better interpreted as stress-induced sensitization of later fear learning. In this sense, SEFL is relevant to generalization and sensitization processes, but it does not cover the full range of fear-generalization phenomena seen in PTSD [6,75].

Sex differences are another important issue. Sanders et al. [76] reported that stress-enhanced fear learning in mice varies with stressor type and sex, with males and females showing different patterns of contextual and tone fear enhancement. Human work points in the same direction: stress effects on fear learning can differ by sex and hormonal status [34,35,77]. In our analysis, the sex subgroup test was not significant (Qb = 0.27, *p* = 0.876), and male-only, female-only, and mixed-sex comparisons all showed positive pooled effects. This null subgroup result should not be taken as evidence that sex is unimportant. The female-only subgroup included only eight comparisons and showed very high heterogeneity (I^2^ = 93.50%), and estrous-cycle information was rarely reported. More balanced inclusion of male and female animals, together with clearer reporting of estrous-cycle or hormonal status, is needed before sex-dependent SEFL effects can be evaluated with confidence.

Finally, the present analysis focused on fear acquisition rather than extinction. This does not mean that extinction is unimportant for SEFL. Impaired extinction and impaired extinction recall are central to many PTSD-relevant accounts of persistent fear [7,39], and SEFL-related procedures could be combined with extinction training and recall tests [10,52]. In the current literature, however, most eligible studies reported freezing during later fear testing but did not provide extractable extinction outcomes. The lack of an extinction analysis should therefore be understood as a scope limitation of this meta-analysis. Future SEFL work that includes extinction, extinction recall, safety learning, or fear-generalization outcomes would help clarify whether prior severe stress affects not only later fear acquisition but also the regulation and reduction of learned fear.

### 4.4. Relationship to Human Stress-Induced Fear-Learning Alterations

Human fear-conditioning studies provide useful but limited background for interpreting SEFL. In trauma-exposed or PTSD samples, previous studies have reported stronger fear learning and greater fear responding during extinction [78]. Other related findings include impaired retrieval of extinction memory [39], disrupted safety-signal learning [79], and biased fear generalization [75]. Human experimental studies also suggest that acute stress can interfere with fear-conditioning processes, including extinction-memory retrieval [80] and fear acquisition when stress is administered shortly before learning [81]. These findings support the need to examine how prior severe stress can influence later fear learning, which is the process that SEFL was designed to examine.

However, human fear-learning studies and rodent SEFL procedures are not directly equivalent. Human studies differ from standard SEFL experiments in stressor type, controllability, stressor intensity, timing between stress exposure and fear learning, outcome measures, and clinical status of the participants. Therefore, the human findings provide translational background for SEFL, but they do not by themselves establish classical construct validity. To make stronger translational claims, future studies would need to bring human and rodent procedures closer together, including comparable stress timing, conditioning design, extinction or safety-learning measures, and intervention effects. Reviews of human stress and fear-conditioning studies also show that stress effects depend on timing, sex, hormones, and task design, which further supports viewing SEFL as a protocol-sensitive paradigm [34].

### 4.5. Intervention-Related Evidence Remains Limited

Predictive validity currently requires a cautious interpretation. Evidence that SEFL outcomes can be changed with pharmacological or behavioral manipulations indicates intervention sensitivity within the paradigm, but this alone does not show that SEFL predicts human treatment response. Intervention-related evidence was sparse among studies eligible for the main synthesis and was too heterogeneous for pooling. Existing studies show that the SEFL phenotype can be modified under some conditions, for example by morphine given within specific post-stress windows [51] or by unconditional stimulus deflation under procedural constraints [82]. These findings indicate intervention sensitivity within the paradigm, but they do not by themselves establish predictive validity in the classical sense. Because these studies differed in design, timing, and endpoint, predictive-validity evidence was summarized qualitatively rather than pooled as a separate effect. Details of the intervention-related feature coding are provided in Supplementary Table S3.

Establishing predictive validity would require standardized SEFL-versus-control intervention studies that test clinically supported PTSD treatments, distinguish prevention from reversal designs, define treatment timing and dose, and report whether such interventions reduce the SEFL phenotype relative to appropriate controls. Existing intervention-related studies are few, differ in timing, manipulation type, and behavioral endpoint, and have not systematically tested standard PTSD pharmacotherapies. In particular, evidence-supported PTSD pharmacotherapies, including selective serotonin reuptake inhibitors (SSRIs) such as sertraline and paroxetine, have not been adequately evaluated in standard SEFL-versus-control designs [83]. Therefore, the current findings should be treated as preliminary intervention-related evidence rather than evidence of established treatment-response validity. Future studies should test clinically relevant treatments within standardized SEFL protocols and evaluate whether animal intervention effects correspond to human treatment responses or to human evidence on stress-induced fear-learning sensitization.

### 4.6. Relation to Other PTSD-Related Animal Models

The comparison with other PTSD-related animal models is mainly useful for setting the boundaries of SEFL. Single prolonged stress (SPS), standard footshock fear-conditioning procedures, social defeat, and early adversity paradigms address related but distinct questions. SPS is usually used for broader multimodal stress questions [84,85,86,87,88]. Standard footshock fear-conditioning procedures offer tighter experimental control because the aversive event can be defined by shock parameters [89,90]. Social defeat, by contrast, places stress in a social context and is more relevant to social threat and avoidance [89,91,92,93,94].

SPS illustrates this distinction clearly. Its multimodal design makes it useful for studying PTSD-related changes in hypothalamic–pituitary–adrenal axis regulation, glucocorticoid feedback, anxiety-like or startle-related behavior, and fear extinction [84,85,86,87,88]. At the same time, this combined-stressor structure limits mechanistic precision, because later outcomes cannot easily be assigned to one component of the stress sequence. SEFL is narrower, but that design makes it more directly aligned with the specific question of stress-induced enhancement of later fear learning [25,26,27].

Standard footshock fear-conditioning procedures and SEFL also differ despite their shared use of footshock. In standard footshock fear-conditioning procedures, the shock usually serves as the aversive event that supports the original fear memory. In SEFL, the initial shock session is used as a prior severe stressor, and the main outcome is later fear learning in a distinct context [11,25,27,52]. This difference in timing and explanatory role is central to why SEFL should be distinguished from standard fear conditioning.

The same point applies to social defeat, learned helplessness, and early adversity, which should be treated as complementary rather than interchangeable with SEFL. Social defeat is more suited to social threat, avoidance, and stress-related social dysfunction [89,91,92,93,94]. Learned helplessness tends to be most informative for questions of controllability, motivation, and the consequences of inescapable stress exposure [19]. Early adversity paradigms are more relevant to developmental vulnerability and later stress-related risk, with human studies linking early-life adversity to later psychopathology [20,21] and rodent models showing long-term effects of early stress exposure [22,23,24]. Although these lines of evidence are useful, they do not isolate the effect of a prior trauma-like exposure on later fear learning in the same way.

Other stress-based models further illustrate why model choice should be question-driven. Restraint or immobilization procedures are easy to implement but vary substantially with duration, repetition, and the degree of movement restriction [17]. Predator-based procedures have ethological relevance and can produce defensive behavior and stress physiology, but standardizing effective threat intensity can be difficult [18]. These models are useful for specific questions, but their endpoints and mechanisms do not map directly onto the SEFL question of later fear-learning sensitization.

Taken together, these comparisons place SEFL most naturally as a focused paradigm for testing whether prior severe stress enhances later fear learning in a separate context.

### 4.7. Boundary Conditions and Limitations of Interpretation

These subgroup patterns should be read in light of the current evidence base, which was dominated by rat and contextual fear-conditioning comparisons, with several smaller subgroups represented by relatively few comparisons. Several subgroup findings should therefore be interpreted as exploratory rather than stable moderator effects, and several methodological issues also limit the strength of the conclusions. Language bias cannot be excluded because the search was restricted to English-language articles. The main quantitative synthesis was restricted to freezing outcomes, so the conclusions mainly apply to fear- and threat-response expression and may not capture other behavioral domains relevant to fear-, anxiety-, trauma-, and stressor-related disorders, such as avoidance, startle, sleep, reward processing, social behavior, or cognition. The certainty of evidence was not assessed using a formal certainty-of-evidence framework, and the descriptive model-relevant feature synthesis should therefore be interpreted descriptively rather than as a formal certainty assessment.

The species and strain findings are one example. They may reflect genuine biological differences, but they are also likely entangled with laboratory-specific protocols, stress intensity, conditioning parameters, and other study-level design features. Exploratory subgroup analysis by species showed positive pooled effects in both rat and mouse comparisons, with a higher pooled estimate in rat comparisons. However, the mouse subgroup included only five comparisons, and several strain subgroups were based on very few comparisons. For this reason, the species and strain results should be treated as exploratory signals rather than firm evidence that particular species or strains are more or less susceptible to SEFL.

Age and developmental stage were also difficult to evaluate. Some included studies used adult animals, whereas others involved early-life or adolescent stress procedures, such as PND17 exposure [53,67,68,70,71]. Where reported, age or developmental stage is summarized in Supplementary Table S2. However, age was not reported consistently and was often linked with other design features, including strain, stress timing, stress type, and testing interval. For this reason, we did not conduct an age-based subgroup analysis. Future studies should report age more precisely and directly test whether SEFL differs across juvenile, adolescent, and adult animals.

A similar caution applies to conditioning modality. Conditioning modality may influence SEFL expression, although the current evidence is limited. Contextual fear conditioning depends strongly on hippocampal contextual processing, whereas auditory/cued fear conditioning relies heavily on amygdala-based cue-shock associations [95,96]. More recent work further implicates anterior cingulate cortex–ventral hippocampal circuits in contextual fear generalization and lateral amygdala plasticity in auditory fear conditioning [97,98]. Both AFC and CFC comparisons showed positive pooled effects, but the between-subgroup difference was not statistically significant and the AFC subgroup included only three comparisons. The present data therefore do not support a definitive conditioning-specific difference, and this absence of a statistically significant subgroup effect should not be interpreted as evidence that AFC and CFC outcomes are equivalent.

Some variation may also reflect features of the behavioral assay itself. Small, near-zero, or occasionally negative effect estimates in some comparisons may partly reflect ceiling effects in freezing. When control animals already show high freezing levels, or when stress and conditioning procedures are highly intense, there may be limited room for additional stress-induced enhancement. On the other hand, weaker SEFL effects may arise when environmental or psychosocial stressors are insufficient to cause lasting sensitization. Testing also should not occur outside of the relevant expression window. The observed pattern in the testing-interval subgroups fits this interpretation of the current review.

Several statistical and design issues should also be considered. Some studies contributed more than one comparison, which may have introduced residual dependence among effect sizes. Although shared control groups were adjusted by dividing the control sample size across relevant comparisons, this approach may not fully account for correlations among comparisons from the same study. The study-level sensitivity analysis yielded a similar pooled estimate, making it unlikely that the overall finding simply reflected studies that contributed several comparisons. Several subgroup strata contained few comparisons; these estimates should therefore be interpreted as exploratory and hypothesis-generating rather than definitive. Multiple subgroup tests were not adjusted, and protocol variables were not independent across studies; therefore, subgroup patterns should be interpreted as signals of boundary conditions rather than causal moderator effects.

Reporting quality was another limitation. The high proportion of unclear ratings across key domains, including allocation concealment, random housing, and blinding of caregivers or investigators, reflects insufficient reporting and reduces confidence in the internal validity of the included studies. Future SEFL studies should follow animal-experiment reporting recommendations such as the Animal Research: Reporting of In Vivo Experiments (ARRIVE) [99] more closely, especially for randomization, blinding, housing, and sample-size justification. Studies would also be easier to compare if they used more standardized stress procedures, reported negative and null findings more transparently, and treated strain and sex as planned experimental factors rather than secondary details.

Funnel-plot asymmetry and Egger’s regression suggested possible small-study effects. Given the substantial heterogeneity, this result should be interpreted cautiously and should not be taken as direct evidence of publication bias. Trim-and-fill imputed 17 comparisons and reduced the pooled estimate, but the adjusted effect remained significant.

### 4.8. Future Directions for SEFL Research

The findings above suggest several practical directions for future SEFL research. Protocol refinement remains important for improving the interpretability and reproducibility of SEFL. Later work has manipulated shock number and intensity to reduce animal burden while preserving the SEFL phenotype [28]. This is consistent with refinement principles in animal research [29,30]. These refinements also help define when SEFL produces durable fear-learning sensitization rather than transient stress reactivity or nonspecific behavioral disruption.

Another useful next step in our investigation would be to compare footshock and non-footshock stressors directly. This would allow us to disentangle the effects of stressor intensity, controllability, and physiological challenge from those of freezing as the main endpoint. Future SEFL studies should also plan species, strain, sex, stress method, conditioning modality, and testing interval as design factors from the outset, rather than treating them as secondary descriptors. This is especially important because several subgroup findings in the present review came from small or confounded categories.

Mechanistic work is also needed to explain why SEFL varies across procedures and testing intervals. Candidate pathways include region- and time-dependent gene regulation in stress-related circuits [100], brain and hippocampal interleukin-1β signaling and dentate gyrus interleukin-1 receptor-dependent stress sensitization [72,101,102], and gastrin-releasing-peptide-related dopamine signaling in the amygdala during SEFL and extinction [103]. These mechanisms were outside the present quantitative synthesis, but they may explain part of the protocol- and time-dependent variation observed here.

Predictive validity remains an open question. A useful next step would be to test clinically relevant interventions in standardized SEFL-versus-control designs. These studies should separate prevention from reversal designs, report treatment timing and dose clearly, and examine whether clinically supported PTSD interventions, including selective serotonin reuptake inhibitors (SSRIs) such as sertraline and paroxetine, can prevent or reverse the SEFL phenotype [83]. Such evidence would make it easier to judge whether treatment effects in SEFL align with human treatment responses or with human findings on stress-induced changes in fear learning.

## 5. Conclusions

The present findings support SEFL as a focused behavioral neuroscience paradigm for studying fear-learning sensitization after severe stress. Across 25 studies and 49 comparisons, prior stress exposure was associated with higher freezing behavior during later fear testing under the REML random-effects model (Hedges’ g = 1.71, 95% CI [1.38, 2.04]). However, the substantial heterogeneity and the 95% prediction interval, which crossed zero, indicate that SEFL should not be expected under every protocol and may be absent or markedly reduced under some experimental conditions. Exploratory between-subgroup variation was observed for several protocol- and sample-level variables, including species, strain, method of stress, testing interval, and stress type. These subgroup findings should be interpreted cautiously, however, because some categories had few comparisons or were confounded with study-level design features. The overall estimate was stable in sensitivity analyses, though some funnel-plot asymmetry and trim-and-fill results indicate that small-study effects may have inflated the pooled estimate.

These findings also define the translational scope of SEFL. The paradigm is most useful for testing whether severe stress in one context enhances later fear learning in another context, but it does not capture PTSD as a whole. The strongest quantitative evidence comes from freezing-defined fear- or threat-response expression, whereas evidence for broader behavioral domains remains limited. Intervention-related evidence remains limited and heterogeneous, and evidence-supported PTSD pharmacotherapies, including selective serotonin reuptake inhibitors (SSRIs) such as sertraline and paroxetine, have not been systematically tested in standard SEFL designs. Therefore, the current intervention-related evidence is not sufficient for a formal judgment about predictive validity. Overall, SEFL is best viewed as a protocol-sensitive paradigm for stress-enhanced later fear learning rather than as a broad PTSD model. The present behavioral estimates may serve as benchmarks for future studies of the neural, molecular, and treatment-related mechanisms of stress-induced learning vulnerability.

## Figures and Tables

**Table 1:** Characteristics of included SEFL comparisons.

Characteristic	Category	Count	Percentage
Included studies	—	25 studies	—
Included comparisons	—	49 comparisons	100%
Publication year	Range	2005–2025	—
Species group	Rat	44	89.8%
	Mouse	5	10.2%
Strain	Long–Evans rats	31	63.3%
	Sprague Dawley rats	8	16.3%
	Wistar rats	1	2.0%
	Wistar Kyoto More Immobile rats	2	4.1%
	Wistar Kyoto Less Immobile rats	2	4.1%
	129S6/SvEvTac mice	1	2.0%
	C57BL/6J mice	4	8.2%
Sex	Male	29	59.2%
	Female	8	16.3%
	Both sexes	12	24.5%
Conditioning type	Contextual fear conditioning	46	93.9%
	Auditory fear conditioning	3	6.1%
Stress type	Acute stress	47	95.9%
	Chronic stress	2	4.1%
Grouped stress method	Classic footshock	29	59.2%
	Chronic footshock	2	4.1%
	Modified footshock	9	18.4%
	Non-footshock stress	9	18.4%
Testing interval	≤3 days	20	40.8%
	4–7 days	5	10.2%
	8–14 days	10	20.4%
	15–30 days	5	10.2%
	>30 days	9	18.4%

Note. Percentages were calculated using 49 comparisons as the denominator, except for the included studies and publication year rows. SEFL = stress-enhanced fear learning.

**Table 2:** Overall and exploratory subgroup analyses using REML random-effects models. Subgroups with small numbers of comparisons should be viewed as hypothesis-generating.

Moderator	Subgroup	k	g	95% CI	I^2^	τ^2^
Overall	All comparisons	49	1.712	[1.382, 2.042]	81.91%	1.072
Species group	Rat	44	1.823	[1.458, 2.188]	80.56%	1.175
	Mouse	5	0.908	[0.577, 1.239]	30.66%	0.043
Strain	129S6/SvEvTac	1	1.536	[0.806, 2.267]	—	0.000
	C57BL/6J	4	0.775	[0.486, 1.064]	0.00%	0.000
	Long–Evans	31	2.104	[1.669, 2.539]	78.75%	1.150
	Sprague Dawley	8	1.667	[1.271, 2.062]	0.00%	0.000
	WLI	2	0.456	[−0.019, 0.931]	0.00%	0.000
	WMI	2	−0.322	[−2.868, 2.223]	94.25%	3.179
	Wistar	1	2.063	[0.820, 3.306]	—	0.000
Grouped stress method	Chronic footshock	2	3.370	[1.932, 4.807]	54.55%	0.595
	Classic footshock	29	1.812	[1.395, 2.229]	77.05%	0.958
	Modified footshock	9	1.955	[1.348, 2.562]	71.17%	0.575
	Non-footshock stress	9	0.816	[0.140, 1.492]	84.74%	0.872
Sex	Both sexes	12	1.826	[1.314, 2.338]	67.76%	0.520
	Female	8	1.486	[0.098, 2.875]	93.50%	3.684
	Male	29	1.707	[1.327, 2.086]	76.61%	0.770
Conditioning type	AFC	3	1.207	[0.342, 2.072]	65.66%	0.381
	CFC	46	1.749	[1.399, 2.099]	81.90%	1.139
Testing interval	≤3 days	20	1.737	[1.099, 2.375]	87.60%	1.788
	4–7 days	5	2.859	[2.019, 3.700]	55.37%	0.496
	8–14 days	10	1.510	[1.096, 1.923]	44.01%	0.179
	15–30 days	5	2.350	[1.097, 3.604]	83.40%	1.610
	>30 days	9	0.930	[0.510, 1.351]	51.48%	0.204
Stress type	Acute stress	47	1.641	[1.315, 1.966]	80.94%	0.985
	Chronic stress	2	3.370	[1.932, 4.807]	54.55%	0.595

Note. k = number of comparisons; g = Hedges’ g; CI = confidence interval; I^2^ = percentage of variability attributable to heterogeneity; REML = restricted maximum likelihood; τ^2^ = between-comparison variance; Qb = between-subgroup heterogeneity statistic; AFC = auditory fear conditioning; CFC = contextual fear conditioning; WLI = Wistar Kyoto Less Immobile rats; WMI = Wistar Kyoto More Immobile rats. All subgroup analyses were exploratory and should not be interpreted as definitive moderator tests. Subgroups with small numbers of comparisons should be interpreted cautiously. Between-subgroup tests were as follows: species group, Qb = 13.27, *p* < 0.001; strain, Qb = 43.65, *p* < 0.001; stress method, Qb = 12.69, *p* = 0.005; sex, Qb = 0.27, *p* = 0.876; conditioning type, Qb = 1.30, *p* = 0.255; testing interval, Qb = 19.41, *p* = 0.001; stress type, Qb = 5.29, *p* = 0.022.

**Table 3:** Sensitivity analyses and assessment of small-study effects.

Analysis	Result	Interpretation
Primary REML random-effects model	Hedges’ g = 1.71, 95% CI [1.38, 2.04], z = 10.16, *p* < 0.001	Large positive overall SEFL effect
95% prediction interval	[−0.40, 3.82]	Crossed zero, indicating that SEFL may be absent or markedly reduced under some experimental settings despite the large average effect
CFC-only sensitivity analysis	k = 46; Hedges’ g = 1.75, 95% CI [1.40, 2.10], z = 9.80, *p* < 0.001	Overall effect remained significant when restricted to contextual fear-conditioning comparisons
Study-level sensitivity analysis	k = 25; Hedges’ g = 1.58, 95% CI [1.22, 1.94], z = 8.54, *p* < 0.001	Overall effect was not solely driven by studies contributing multiple comparisons
Excluding non-footshock stress	k = 40; Hedges’ g = 1.92, 95% CI [1.57, 2.27], z = 10.89, *p* < 0.001	Overall effect remained significant after excluding non-footshock stress comparisons
Leave-one-out sensitivity analysis	Hedges’ g ranged from 1.647 to 1.759; all 95% CIs remained above zero	Overall effect was not driven by any single comparison
Egger’s regression test	β = 5.60, SE = 0.694, z = 8.07, *p* < 0.001	Suggests possible small-study effects
Trim-and-fill analysis	17 comparisons imputed; adjusted Hedges’ g = 1.10, 95% CI [0.71, 1.49]	Adjusted estimate was smaller but remained statistically significant

Note. REML = restricted maximum likelihood; CFC = contextual fear conditioning; CI = confidence interval; SE = standard error; SEFL = stress-enhanced fear learning. β denotes the Egger regression coefficient. Effect estimates, confidence intervals, and prediction intervals are rounded for presentation.

## Data Availability

The extracted data supporting the findings of this meta-analysis are available from the corresponding author upon reasonable request. Summary data are reported in the manuscript and Supplementary Materials.

## Supplementary Materials

The following supporting information can be downloaded at: https://www.mdpi.com/article/10.3390/brainsci16070691/s1, Supplementary File S1: completed PRISMA 2020 checklist; Supplementary Methods: complete original and expanded search strategies; Table S1: excluded full-text studies with reasons; Table S2: comparison-level extraction data, including age/developmental-stage information, parent study labels, and shared-control adjustments; Table S3: study-level model-relevant feature coding, including intervention-related evidence; Table S4: study-level SYRCLE risk-of-bias judgments; Table S5: leave-one-out sensitivity analysis; Table S6: exploratory subgroup analyses; Figure S1: SYRCLE risk-of-bias assessment, including the risk-of-bias graph and study-level summary; Figures S2–S8: subgroup forest plots; Figure S9: leave-one-out sensitivity analysis; Figure S10: funnel plot; Figure S11: study-level sensitivity analysis; Figure S12: forest plot after excluding non-footshock stressor comparisons.

## Abbreviations

The following abbreviations are used in this manuscript:

PTSD	Posttraumatic stress disorder
SEFL	Stress-enhanced fear learning
SPS	Single prolonged stress
CFC	Contextual fear conditioning
AFC	Auditory fear conditioning
REML	Restricted maximum likelihood
CI	Confidence interval
PRISMA	Preferred Reporting Items for Systematic Reviews and Meta-Analyses
SYRCLE	Systematic Review Centre for Laboratory Animal Experimentation
SSRIs	Selective serotonin reuptake inhibitors
