# Supplementary material for: Stress-Enhanced Fear Learning in Rodents: A Systematic Review and Meta-Analysis of Fear-Learning Sensitization After Severe Stress
Source: Brain Sci. 2026 Jun 30;16(7):691. doi: 10.3390/brainsci16070691 (PMC13406739; doi:10.3390/brainsci16070691)
Supplement: Supplementary file 1 [file brainsci-16-00691-s001.zip › Supplementary_Figures_SEFL.pdf]

Supplementary Figures

Supplementary figures for: Stress-Enhanced Fear Learning in Rodents: A Systematic Review and Meta-Analysis of Fear-Learning Sensitization After Severe Stress

Figure S1. SYRCLE risk-of-bias summary.

(A) Risk-of-bias graph.

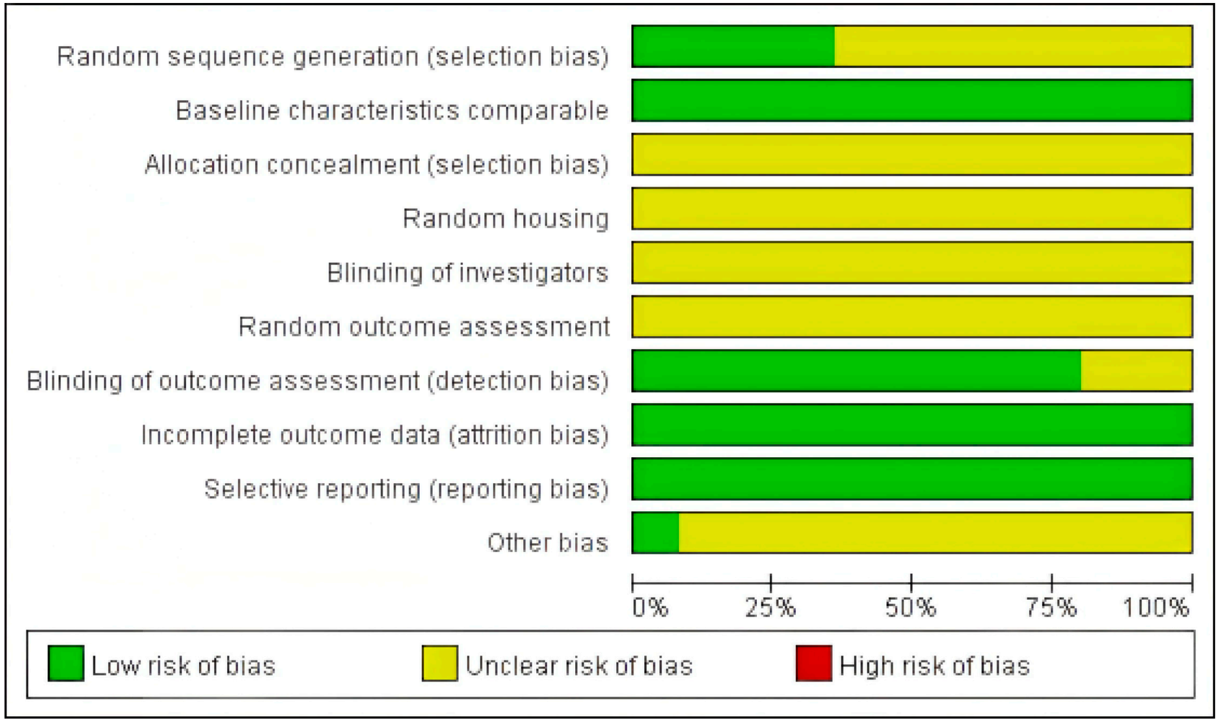

Figure S1. Continued.

(B) Study-level risk-of-bias summary.

	Random sequence generation (selection bias)	Baseline characteristics comparable	Allocation concealment (selection bias)	Random housing	Blinding of investigators	Random outcome assessment	Blinding of outcome assessment (detection bias)	Incomplete outcome data (attrition bias)	Selective reporting (reporting bias)	Other bias
Asch, 2023	+	+	?	?	?	?	+	+	+	?
Conoscenti, 2022	+	+	?	?	?	?	+	+	+	+
Conoscenti, 2024	?	+	?	?	?	?	+	+	+	+
Deschaux, 2015	+	+	?	?	?	?	?	+	+	?
Hassien, 2020	?	+	?	?	?	?	+	+	+	?
Hersman, 2019	?	+	?	?	?	?	+	+	+	?
Jeong, 2020	?	+	?	?	?	?	+	+	+	?
Jones, 2015	?	+	?	?	?	?	+	+	+	?
Long, 2012	+	+	?	?	?	?	+	+	+	?
Lopez-Kawa, 2025	?	+	?	?	?	?	+	+	+	?
Meyer, 2013	+	+	?	?	?	?	+	+	+	?
Minshall, 2024a	?	+	?	?	?	?	+	+	+	?
Minshall, 2024b	?	+	?	?	?	?	+	+	+	?
Minshall, 2025	?	+	?	?	?	?	+	+	+	?
Parekh, 2021	+	+	?	?	?	?	+	+	+	?
Pennington, 2020	?	+	?	?	?	?	?	+	+	?
Perusini, 2016	+	+	?	?	?	?	+	+	+	?
Pizzimenti, 2017	?	+	?	?	?	?	?	+	+	?
Ponomarev, 2010	+	+	?	?	?	?	?	+	+	?
Poulos, 2015	?	+	?	?	?	?	+	+	+	?
Przybyl, 2021	?	+	?	?	?	?	+	+	+	?
Quinn, 2014	?	+	?	?	?	?	+	+	+	?
Rau, 2005	+	+	?	?	?	?	?	+	+	?
Sneddon, 2021	?	+	?	?	?	?	+	+	+	?
Szczytkowski-Thomson, 2013	?	+	?	?	?	?	+	+	+	?

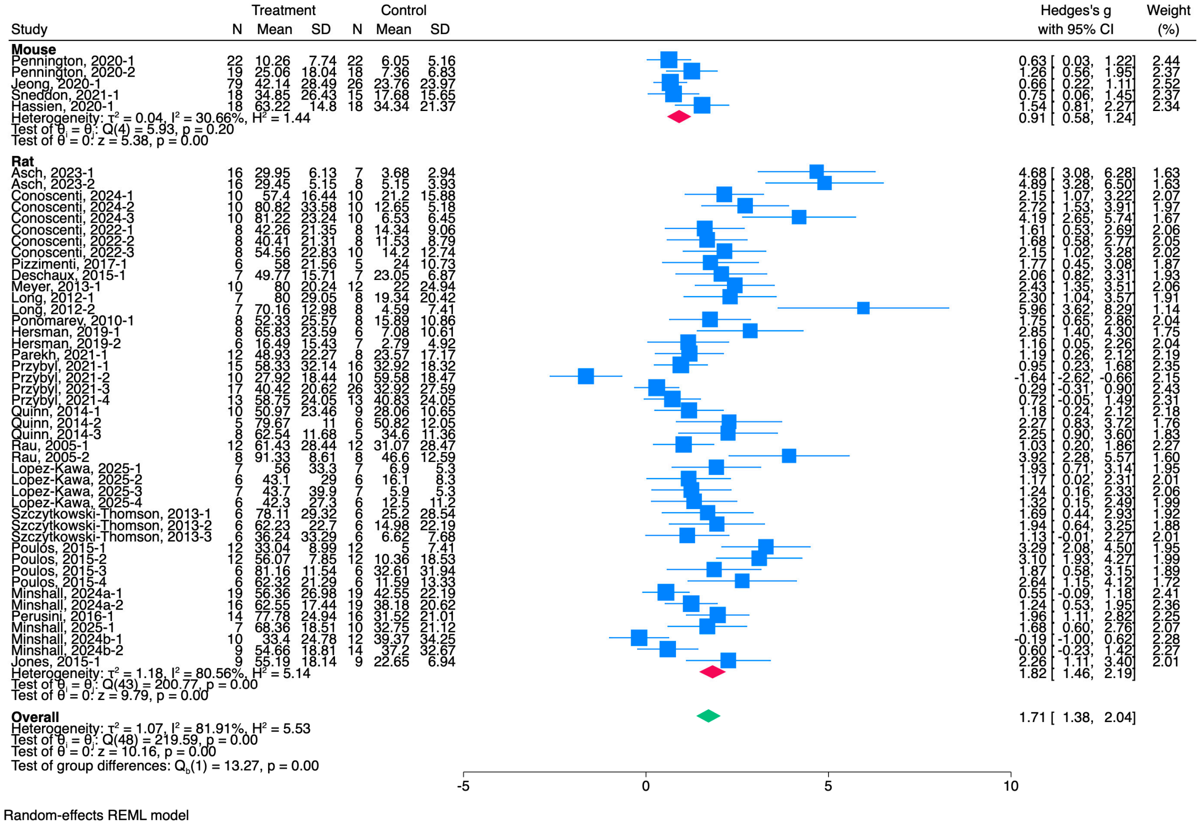

Figure S2. Subgroup forest plot by species.

Study	Treatment N	Treatment Mean	Treatment SD	Control N	Control Mean	Control SD	Hedges's g with 95% CI	Weight (%)
**129S6/SvEvTac**								
Hassien, 2020-1	18	63.22	14.8	18	34.34	21.37	1.54 [0.81, 2.27]	2.34
Heterogeneity: $\tau^2 = 0.00$, I^2 = .%, H^2 = .							1.54 [0.81, 2.27]	
Test of $\theta_i = \theta_j$: Q(0) = 0.00, p = .								
Test of $\theta = 0$: z = 4.12, p = 0.00								
**C57BL/6J**								
Pennington, 2020-1	22	10.26	7.74	22	6.05	5.16	0.63 [0.03, 1.22]	2.44
Pennington, 2020-2	19	25.06	18.04	18	7.36	6.83	1.26 [0.56, 1.95]	2.37
Jeong, 2020-1	79	42.14	28.49	26	23.76	23.97	0.66 [0.22, 1.11]	2.52
Sneddon, 2021-1	18	34.85	26.43	15	17.68	15.65	0.75 [0.06, 1.45]	2.37
Heterogeneity: $\tau^2 = 0.00$, $I^2 = 0.00\%$, $H^2 = 1.00$							0.77 [0.49, 1.06]	
Test of $\theta_i = \theta_j$: Q(3) = 2.32, p = 0.51								
Test of $\theta = 0$: z = 5.25, p = 0.00								
**Long-Evans**								
Asch, 2023-1	16	29.95	6.13	7	3.68	2.94	4.68 [3.08, 6.28]	1.63
Asch, 2023-2	16	29.45	5.15	8	5.15	3.93	4.89 [3.28, 6.50]	1.63
Conoscenti, 2024-1	10	57.4	16.44	10	21.2	15.88	2.15 [1.07, 3.22]	2.07
Conoscenti, 2024-2	10	80.82	33.58	10	12.65	5.18	2.72 [1.53, 3.91]	1.97
Conoscenti, 2024-3	10	81.22	23.24	10	6.53	6.45	4.19 [2.65, 5.74]	1.67
Pizzimenti, 2017-1	6	58	21.56	5	24	10.73	1.77 [0.45, 3.08]	1.87
Meyer, 2013-1	10	80	20.24	12	22	24.94	2.43 [1.35, 3.51]	2.06
Long, 2012-1	7	80	29.05	8	19.34	20.42	2.30 [1.04, 3.57]	1.91
Long, 2012-2	7	70.16	12.98	8	4.59	7.41	5.96 [3.62, 8.29]	1.14
Ponomarev, 2010-1	8	52.33	25.57	8	15.89	10.86	1.75 [0.65, 2.86]	2.04
Hersman, 2019-1	8	65.83	23.59	6	7.08	10.61	2.85 [1.40, 4.30]	1.75
Hersman, 2019-2	6	16.49	15.43	7	2.79	4.92	1.16 [0.05, 2.26]	2.04
Quinn, 2014-1	10	50.97	23.46	9	28.06	10.65	1.18 [0.24, 2.12]	2.18
Quinn, 2014-2	5	79.67	11	6	50.82	12.05	2.27 [0.83, 3.72]	1.76
Quinn, 2014-3	8	62.54	11.68	5	34.6	11.36	2.25 [0.90, 3.60]	1.83
Rau, 2005-1	12	61.43	28.44	12	31.07	28.47	1.03 [0.20, 1.86]	2.27
Rau, 2005-2	8	91.33	8.61	8	46.6	12.59	3.92 [2.28, 5.57]	1.60
Lopez-Kawa, 2025-1	7	56	33.3	7	6.9	5.3	1.93 [0.71, 3.14]	1.95
Lopez-Kawa, 2025-2	6	43.1	29	6	16.1	8.3	1.17 [0.02, 2.31]	2.01
Lopez-Kawa, 2025-3	7	43.7	39.9	7	5.9	5.3	1.24 [0.16, 2.33]	2.06
Lopez-Kawa, 2025-4	6	42.3	27.3	6	12.5	11.2	1.32 [0.15, 2.49]	1.99
Poulos, 2015-1	12	33.04	8.99	12	5	7.41	3.29 [2.08, 4.50]	1.95
Poulos, 2015-2	12	56.07	7.85	12	10.36	18.53	3.10 [1.93, 4.27]	1.99
Poulos, 2015-3	6	81.16	11.54	6	32.61	31.94	1.87 [0.58, 3.15]	1.89
Poulos, 2015-4	6	62.32	21.29	6	11.59	13.33	2.64 [1.15, 4.12]	1.72
Minshall, 2024a-1	19	56.36	26.98	19	42.55	22.19	0.55 [-0.09, 1.18]	2.41
Minshall, 2024a-2	16	62.55	17.44	19	38.18	20.62	1.24 [0.53, 1.95]	2.36
Perusini, 2016-1	14	77.78	24.94	16	31.52	21.01	1.96 [1.11, 2.82]	2.25
Minshall, 2025-1	7	68.36	18.51	10	32.75	21.12	1.68 [0.60, 2.76]	2.07
Minshall, 2024b-1	10	33.4	24.78	12	39.37	34.25	-0.19 [-1.00, 0.62]	2.28
Minshall, 2024b-2	9	54.66	18.81	14	37.2	32.67	0.60 [-0.23, 1.42]	2.27
Heterogeneity: $\tau^2 = 1.15$, $I^2 = 78.75\%$, $H^2 = 4.71$							2.10 [1.67, 2.54]	
Test of $\theta_i = \theta_j$: Q(30) = 130.88, p = 0.00								
Test of $\theta = 0$: z = 9.47, p = 0.00								
**Sprague-Dawley**								
Conoscenti, 2022-1	8	42.26	21.35	8	14.34	9.06	1.61 [0.53, 2.69]	2.06
Conoscenti, 2022-2	8	40.41	21.31	8	11.53	8.79	1.68 [0.58, 2.77]	2.05
Conoscenti, 2022-3	8	54.56	22.83	10	14.2	12.74	2.15 [1.02, 3.28]	2.02
Parekh, 2021-1	12	48.93	22.27	8	23.57	17.17	1.19 [0.26, 2.12]	2.19
Szczytkowski-Thomson, 2013-1	6	78.11	29.32	6	25.2	28.54	1.69 [0.44, 2.93]	1.92
Szczytkowski-Thomson, 2013-2	6	62.23	22.7	6	14.98	22.19	1.94 [0.64, 3.25]	1.88
Szczytkowski-Thomson, 2013-3	6	36.24	33.29	6	6.62	7.68	1.13 [-0.01, 2.27]	2.01
Jones, 2015-1	9	55.19	18.14	9	22.65	6.94	2.26 [1.11, 3.40]	2.01
Heterogeneity: $\tau^2 = 0.00$, $I^2 = 0.00\%$, $H^2 = 1.00$							1.67 [1.27, 2.06]	
Test of $\theta_i = \theta_j$: Q(7) = 3.76, p = 0.81								
Test of $\theta = 0$: z = 8.26, p = 0.00								
**WLI**								
Przybyl, 2021-3	17	40.42	20.62	26	32.92	27.59	0.29 [-0.31, 0.90]	2.43
Przybyl, 2021-4	13	58.75	24.05	13	40.83	24.05	0.72 [-0.05, 1.49]	2.31
Heterogeneity: $\tau^2 = 0.00$, $I^2 = 0.00\%$, $H^2 = 1.00$							0.46 [-0.02, 0.93]	
Test of $\theta_i = \theta_j$: Q(1) = 0.74, p = 0.39								
Test of $\theta = 0$: z = 1.88, p = 0.06								
**WMI**								
Przybyl, 2021-1	15	58.33	32.14	16	32.92	18.32	0.95 [0.23, 1.68]	2.35
Przybyl, 2021-2	10	27.92	18.44	10	59.58	18.47	-1.64 [-2.62, -0.66]	2.15
Heterogeneity: $\tau^2 = 3.18$, $I^2 = 94.25\%$, $H^2 = 17.38$							-0.32 [-2.87, 2.22]	
Test of $\theta_i = \theta_j$: Q(1) = 17.38, p = 0.00								
Test of $\theta = 0$: z = -0.25, p = 0.80								
**Wistar**								
Deschaux, 2015-1	7	49.77	15.71	7	23.05	6.87	2.06 [0.82, 3.31]	1.93
Heterogeneity: $\tau^2 = 0.00$, I^2 = .%, H^2 = .							2.06 [0.82, 3.31]	
Test of $\theta_i = \theta_j$: Q(0) = 0.00, p = .								
Test of $\theta = 0$: z = 3.25, p = 0.00								
**Overall**							1.71 [1.38, 2.04]	
Heterogeneity: $\tau^2 = 1.07$, $I^2 = 81.91\%$, $H^2 = 5.53$								
Test of $\theta_i = \theta_j$: Q(48) = 219.59, p = 0.00								
Test of $\theta = 0$: z = 10.16, p = 0.00								
Test of group differences: $Q_b(6)$ = 43.65, p = 0.00								

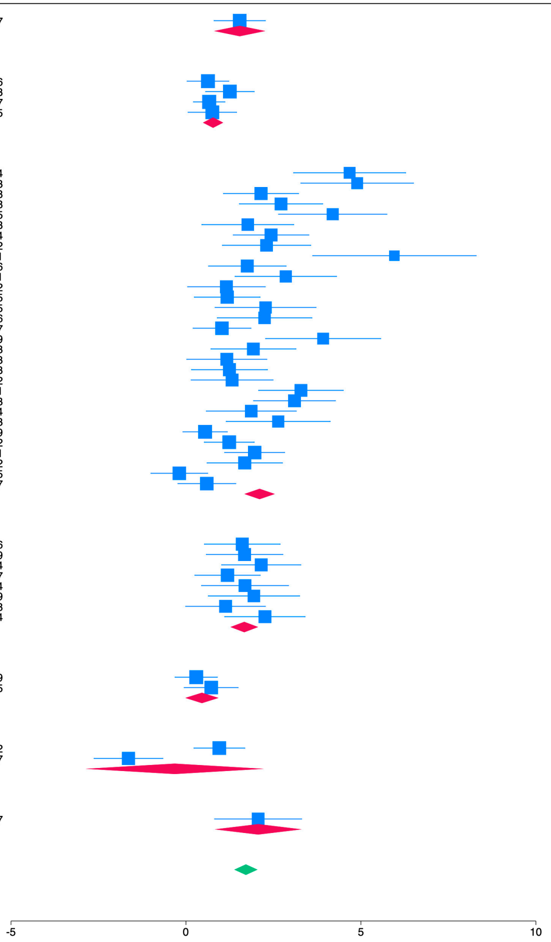

Random-effects REML model

Figure S3. Subgroup forest plot by strain.

Study	Treatment N	Treatment Mean	Treatment SD	Control N	Control Mean	Control SD	Hedges's g with 95% CI	Weight (%)
**Both sexes**								
Conoscenti, 2024-1	10	57.4	16.44	10	21.2	15.88	2.15 [1.07, 3.22]	2.07
Pennington, 2020-1	22	10.26	7.74	22	6.05	5.16	0.63 [0.03, 1.22]	2.44
Quinn, 2014-1	10	50.97	23.46	9	28.06	10.65	1.18 [0.24, 2.12]	2.18
Quinn, 2014-2	5	79.67	11	6	50.82	12.05	2.27 [0.83, 3.72]	1.76
Quinn, 2014-3	8	62.54	11.68	5	34.6	11.36	2.25 [0.90, 3.60]	1.83
Poulos, 2015-1	12	33.04	8.99	12	5	7.41	3.29 [2.08, 4.50]	1.95
Poulos, 2015-2	12	56.07	7.85	12	10.36	18.53	3.10 [1.93, 4.27]	1.99
Poulos, 2015-3	6	81.16	11.54	6	32.61	31.94	1.87 [0.58, 3.15]	1.89
Poulos, 2015-4	6	62.32	21.29	6	11.59	13.33	2.64 [1.15, 4.12]	1.72
Sneddon, 2021-1	18	34.85	26.43	15	17.68	15.65	0.75 [0.06, 1.45]	2.37
Hassien, 2020-1	18	63.22	14.8	18	34.34	21.37	1.54 [0.81, 2.27]	2.34
Minshall, 2025-1	7	68.36	18.51	10	32.75	21.12	1.68 [0.60, 2.76]	2.07
Heterogeneity: $\tau^2 = 0.52$, $I^2 = 67.76\%$, $H^2 = 3.10$							1.83 [1.31, 2.34]	
Test of $\theta_i = \theta_j$: Q(11) = 35.07, p = 0.00								
Test of $\theta = 0$: z = 6.99, p = 0.00								
**Female**								
Asch, 2023-2	16	29.45	5.15	8	5.15	3.93	4.89 [3.28, 6.50]	1.63
Conoscenti, 2024-3	10	81.22	23.24	10	6.53	6.45	4.19 [2.65, 5.74]	1.67
Przybyl, 2021-2	10	27.92	18.44	10	59.58	18.47	-1.64 [-2.62, -0.66]	2.15
Przybyl, 2021-4	13	58.75	24.05	13	40.83	24.05	0.72 [-0.05, 1.49]	2.31
Lopez-Kawa, 2025-2	6	43.1	29	6	16.1	8.3	1.17 [0.02, 2.31]	2.01
Lopez-Kawa, 2025-4	6	42.3	27.3	6	12.5	11.2	1.32 [0.15, 2.49]	1.99
Minshall, 2024a-2	16	62.55	17.44	19	38.18	20.62	1.24 [0.53, 1.95]	2.36
Minshall, 2024b-2	9	54.66	18.81	14	37.2	32.67	0.60 [-0.23, 1.42]	2.27
Heterogeneity: $\tau^2 = 3.68$, $I^2 = 93.50\%$, $H^2 = 15.39$							1.49 [0.10, 2.87]	
Test of $\theta_i = \theta_j$: Q(7) = 68.91, p = 0.00								
Test of $\theta = 0$: z = 2.10, p = 0.04								
**Male**								
Asch, 2023-1	16	29.95	6.13	7	3.68	2.94	4.68 [3.08, 6.28]	1.63
Conoscenti, 2024-2	10	80.82	33.58	10	12.65	5.18	2.72 [1.53, 3.91]	1.97
Conoscenti, 2022-1	8	42.26	21.35	8	14.34	9.06	1.61 [0.53, 2.69]	2.06
Conoscenti, 2022-2	8	40.41	21.31	8	11.53	8.79	1.68 [0.58, 2.77]	2.05
Conoscenti, 2022-3	8	54.56	22.83	10	14.2	12.74	2.15 [1.02, 3.28]	2.02
Pizzimenti, 2017-1	6	58	21.56	5	24	10.73	1.77 [0.45, 3.08]	1.87
Deschaux, 2015-1	7	49.77	15.71	7	23.05	6.87	2.06 [0.82, 3.31]	1.93
Meyer, 2013-1	10	80	20.24	12	22	24.94	2.43 [1.35, 3.51]	2.06
Long, 2012-1	7	80	29.05	8	19.34	20.42	2.30 [1.04, 3.57]	1.91
Long, 2012-2	7	70.16	12.98	8	4.59	7.41	5.96 [3.62, 8.29]	1.14
Ponomarev, 2010-1	8	52.33	25.57	8	15.89	10.86	1.75 [0.65, 2.86]	2.04
Hersman, 2019-1	8	65.83	23.59	6	7.08	10.61	2.85 [1.40, 4.30]	1.75
Hersman, 2019-2	6	16.49	15.43	7	2.79	4.92	1.16 [0.05, 2.26]	2.04
Pennington, 2020-2	19	25.06	18.04	18	7.36	6.83	1.26 [0.56, 1.95]	2.37
Parekh, 2021-1	12	48.93	22.27	8	23.57	17.17	1.19 [0.26, 2.12]	2.19
Jeong, 2020-1	79	42.14	28.49	26	23.76	23.97	0.66 [0.22, 1.11]	2.52
Przybyl, 2021-1	15	58.33	32.14	16	32.92	18.32	0.95 [0.23, 1.68]	2.35
Przybyl, 2021-3	17	40.42	20.62	26	32.92	27.59	0.29 [-0.31, 0.90]	2.43
Rau, 2005-1	12	61.43	28.44	12	31.07	28.47	1.03 [0.20, 1.86]	2.27
Rau, 2005-2	8	91.33	8.61	8	46.6	12.59	3.92 [2.28, 5.57]	1.60
Lopez-Kawa, 2025-1	7	56	33.3	7	6.9	5.3	1.93 [0.71, 3.14]	1.95
Lopez-Kawa, 2025-3	7	43.7	39.9	7	5.9	5.3	1.24 [0.16, 2.33]	2.06
Szczytkowski-Thomson, 2013-1	6	78.11	29.32	6	25.2	28.54	1.69 [0.44, 2.93]	1.92
Szczytkowski-Thomson, 2013-2	6	62.23	22.7	6	14.98	22.19	1.94 [0.64, 3.25]	1.88
Szczytkowski-Thomson, 2013-3	6	36.24	33.29	6	6.62	7.68	1.13 [-0.01, 2.27]	2.01
Minshall, 2024a-1	19	56.36	26.98	19	42.55	22.19	0.55 [-0.09, 1.18]	2.41
Perusini, 2016-1	14	77.78	24.94	16	31.52	21.01	1.96 [1.11, 2.82]	2.25
Minshall, 2024b-1	10	33.4	24.78	12	39.37	34.25	-0.19 [-1.00, 0.62]	2.28
Jones, 2015-1	9	55.19	18.14	9	22.65	6.94	2.26 [1.11, 3.40]	2.01
Heterogeneity: $\tau^2 = 0.77$, $I^2 = 76.61\%$, $H^2 = 4.27$							1.71 [1.33, 2.09]	
Test of $\theta_i = \theta_j$: Q(28) = 109.82, p = 0.00								
Test of $\theta = 0$: z = 8.82, p = 0.00								
**Overall**							1.71 [1.38, 2.04]	
Heterogeneity: $\tau^2 = 1.07$, $I^2 = 81.91\%$, $H^2 = 5.53$								
Test of $\theta_i = \theta_j$: Q(48) = 219.59, p = 0.00								
Test of $\theta = 0$: z = 10.16, p = 0.00								
Test of group differences: $Q_b(2) = 0.27$, p = 0.88								

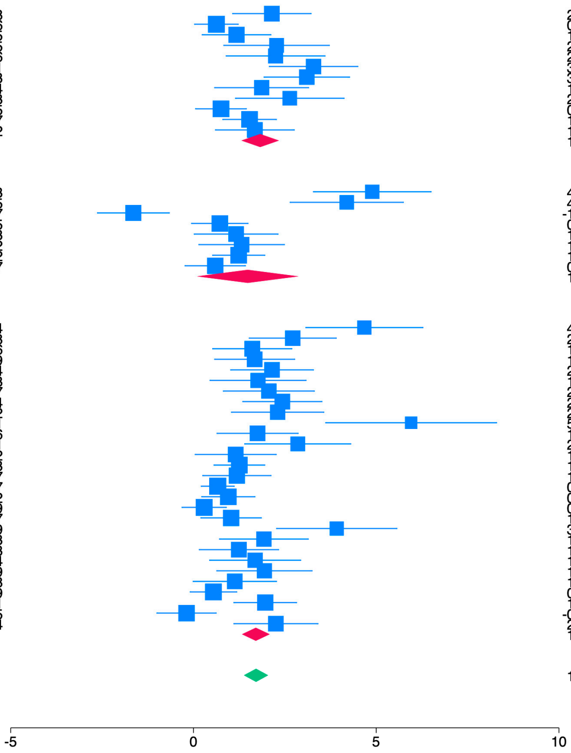

Random-effects REML model

Figure S4. Subgroup forest plot by sex.

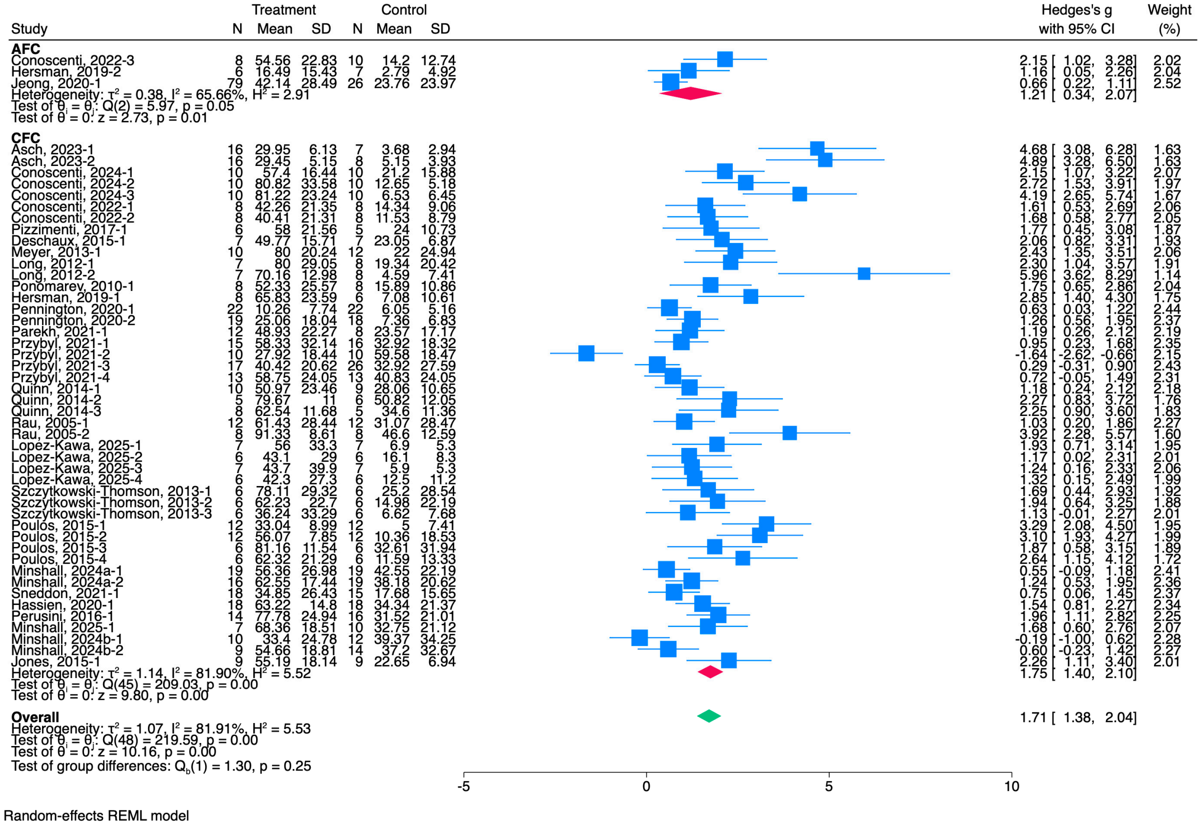

Figure S5. Subgroup forest plot by conditioning type.

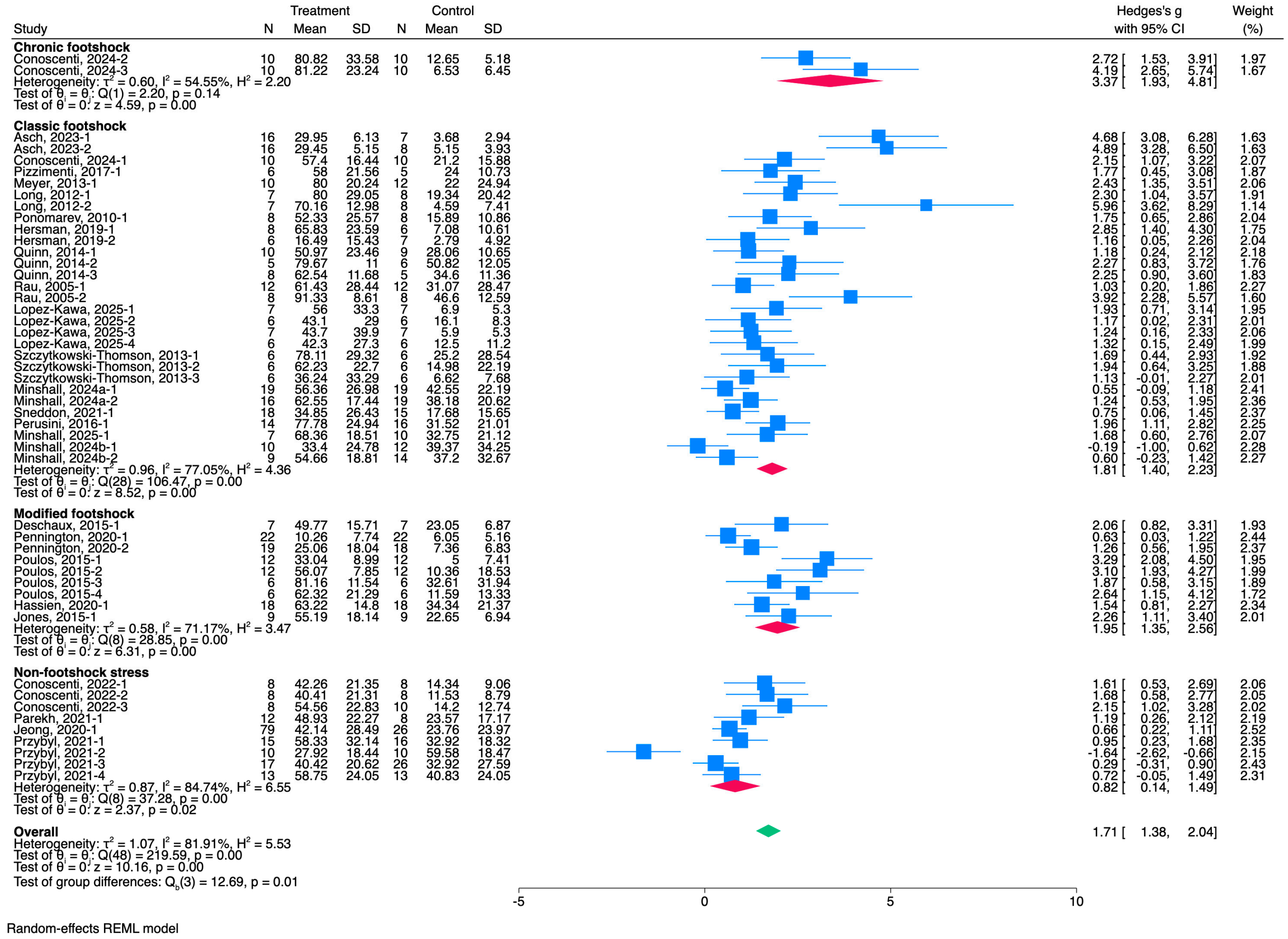

Figure S6. Subgroup forest plot by stress method.

Study	Treatment N	Treatment Mean	Treatment SD	Control N	Control Mean	Control SD	Hedges's g with 95% CI	Weight (%)
**<=3 days**								
Asch, 2023-1	16	29.95	6.13	7	3.68	2.94	4.68 [3.08, 6.28]	1.63
Asch, 2023-2	16	29.45	5.15	8	5.15	3.93	4.89 [3.28, 6.50]	1.63
Conoscenti, 2022-1	8	42.26	21.35	8	14.34	9.06	1.61 [0.53, 2.69]	2.06
Pizzimenti, 2017-1	6	58	21.56	5	24	10.73	1.77 [0.45, 3.08]	1.87
Meyer, 2013-1	10	80	20.24	12	22	24.94	2.43 [1.35, 3.51]	2.06
Hersman, 2019-1	8	65.83	23.59	6	7.08	10.61	2.85 [1.40, 4.30]	1.75
Hersman, 2019-2	6	16.49	15.43	7	2.79	4.92	1.16 [0.05, 2.26]	2.04
Pennington, 2020-1	22	10.26	7.74	22	6.05	5.16	0.63 [0.03, 1.22]	2.44
Przybyl, 2021-1	15	58.33	32.14	16	32.92	18.32	0.95 [0.23, 1.68]	2.35
Przybyl, 2021-2	10	27.92	18.44	10	59.58	18.47	-1.64 [-2.62, -0.66]	2.15
Przybyl, 2021-3	17	40.42	20.62	26	32.92	27.59	0.29 [-0.31, 0.90]	2.43
Przybyl, 2021-4	13	58.75	24.05	13	40.83	24.05	0.72 [-0.05, 1.49]	2.31
Quinn, 2014-1	10	50.97	23.46	9	28.06	10.65	1.18 [0.24, 2.12]	2.18
Rau, 2005-1	12	61.43	28.44	12	31.07	28.47	1.03 [0.20, 1.86]	2.27
Lopez-Kawa, 2025-1	7	56	33.3	7	6.9	5.3	1.93 [0.71, 3.14]	1.95
Lopez-Kawa, 2025-2	6	43.1	29	6	16.1	8.3	1.17 [0.02, 2.31]	2.01
Poulos, 2015-1	12	33.04	8.99	12	5	7.41	3.29 [2.08, 4.50]	1.95
Poulos, 2015-2	12	56.07	7.85	12	10.36	18.53	3.10 [1.93, 4.27]	1.99
Poulos, 2015-3	6	81.16	11.54	6	32.61	31.94	1.87 [0.58, 3.15]	1.89
Poulos, 2015-4	6	62.32	21.29	6	11.59	13.33	2.64 [1.15, 4.12]	1.72
Heterogeneity: $\tau^2 = 1.79$, $I^2 = 87.60\%$, $H^2 = 8.07$							1.74 [1.10, 2.37]	
Test of $\theta_i = \theta_j$: Q(19) = 123.61, p = 0.00								
Test of $\theta = 0$: z = 5.34, p = 0.00								
**4-7 days**								
Conoscenti, 2024-2	10	80.82	33.58	10	12.65	5.18	2.72 [1.53, 3.91]	1.97
Conoscenti, 2024-3	10	81.22	23.24	10	6.53	6.45	4.19 [2.65, 5.74]	1.67
Long, 2012-1	7	80	29.05	8	19.34	20.42	2.30 [1.04, 3.57]	1.91
Rau, 2005-2	8	91.33	8.61	8	46.6	12.59	3.92 [2.28, 5.57]	1.60
Perusini, 2016-1	14	77.78	24.94	16	31.52	21.01	1.96 [1.11, 2.82]	2.25
Heterogeneity: $\tau^2 = 0.50$, $I^2 = 55.37\%$, $H^2 = 2.24$							2.86 [2.02, 3.70]	
Test of $\theta_i = \theta_j$: Q(4) = 8.89, p = 0.06								
Test of $\theta = 0$: z = 6.67, p = 0.00								
**8-14 days**								
Conoscenti, 2022-2	8	40.41	21.31	8	11.53	8.79	1.68 [0.58, 2.77]	2.05
Conoscenti, 2022-3	8	54.56	22.83	10	14.2	12.74	2.15 [1.02, 3.28]	2.02
Parekh, 2021-1	12	48.93	22.27	8	23.57	17.17	1.19 [0.26, 2.12]	2.19
Jeong, 2020-1	79	42.14	28.49	26	23.76	23.97	0.66 [0.22, 1.11]	2.52
Quinn, 2014-2	5	79.67	11	6	50.82	12.05	2.27 [0.83, 3.72]	1.76
Szczytkowski-Thomson, 2013-1	6	78.11	29.32	6	25.2	28.54	1.69 [0.44, 2.93]	1.92
Szczytkowski-Thomson, 2013-2	6	62.23	22.7	6	14.98	22.19	1.94 [0.64, 3.25]	1.88
Szczytkowski-Thomson, 2013-3	6	36.24	33.29	6	6.62	7.68	1.13 [-0.01, 2.27]	2.01
Hassien, 2020-1	18	63.22	14.8	18	34.34	21.37	1.54 [0.81, 2.27]	2.34
Jones, 2015-1	9	55.19	18.14	9	22.65	6.94	2.26 [1.11, 3.40]	2.01
Heterogeneity: $\tau^2 = 0.18$, $I^2 = 44.01\%$, $H^2 = 1.79$							1.51 [1.10, 1.92]	
Test of $\theta_i = \theta_j$: Q(9) = 16.59, p = 0.06								
Test of $\theta = 0$: z = 7.16, p = 0.00								
**15-30 days**								
Conoscenti, 2024-1	10	57.4	16.44	10	21.2	15.88	2.15 [1.07, 3.22]	2.07
Deschaux, 2015-1	7	49.77	15.71	7	23.05	6.87	2.06 [0.82, 3.31]	1.93
Long, 2012-2	7	70.16	12.98	8	4.59	7.41	5.96 [3.62, 8.29]	1.14
Ponomarev, 2010-1	8	52.33	25.57	8	15.89	10.86	1.75 [0.65, 2.86]	2.04
Pennington, 2020-2	19	25.06	18.04	18	7.36	6.83	1.26 [0.56, 1.95]	2.37
Heterogeneity: $\tau^2 = 1.61$, $I^2 = 83.40\%$, $H^2 = 6.02$							2.35 [1.10, 3.60]	
Test of $\theta_i = \theta_j$: Q(4) = 15.11, p = 0.00								
Test of $\theta = 0$: z = 3.68, p = 0.00								
**>30 days**								
Quinn, 2014-3	8	62.54	11.68	5	34.6	11.36	2.25 [0.90, 3.60]	1.83
Lopez-Kawa, 2025-3	7	43.7	39.9	7	5.9	5.3	1.24 [0.16, 2.33]	2.06
Lopez-Kawa, 2025-4	6	42.3	27.3	6	12.5	11.2	1.32 [0.15, 2.49]	1.99
Minshall, 2024a-1	19	56.36	26.98	19	42.55	22.19	0.55 [-0.09, 1.18]	2.41
Minshall, 2024a-2	16	62.55	17.44	19	38.18	20.62	1.24 [0.53, 1.95]	2.36
Sneddon, 2021-1	18	34.85	26.43	15	17.68	15.65	0.75 [0.06, 1.45]	2.37
Minshall, 2025-1	7	68.36	18.51	10	32.75	21.12	1.68 [0.60, 2.76]	2.07
Minshall, 2024b-1	10	33.4	24.78	12	39.37	34.25	-0.19 [-1.00, 0.62]	2.28
Minshall, 2024b-2	9	54.66	18.81	14	37.2	32.67	0.60 [-0.23, 1.42]	2.27
Heterogeneity: $\tau^2 = 0.20$, $I^2 = 51.48\%$, $H^2 = 2.06$							0.93 [0.51, 1.35]	
Test of $\theta_i = \theta_j$: Q(8) = 16.30, p = 0.04								
Test of $\theta = 0$: z = 4.34, p = 0.00								
**Overall**							1.71 [1.38, 2.04]	
Heterogeneity: $\tau^2 = 1.07$, $I^2 = 81.91\%$, $H^2 = 5.53$								
Test of $\theta_i = \theta_j$: Q(48) = 219.59, p = 0.00								
Test of $\theta = 0$: z = 10.16, p = 0.00								
Test of group differences: $Q_b(4) = 19.41$, p = 0.00								

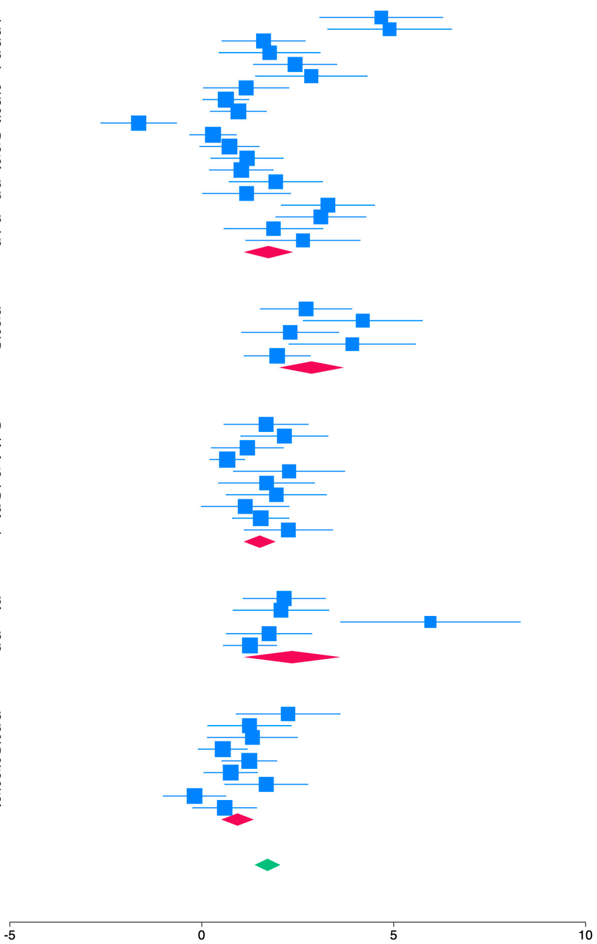

Random-effects REML model

Figure S7. Subgroup forest plot by testing interval.

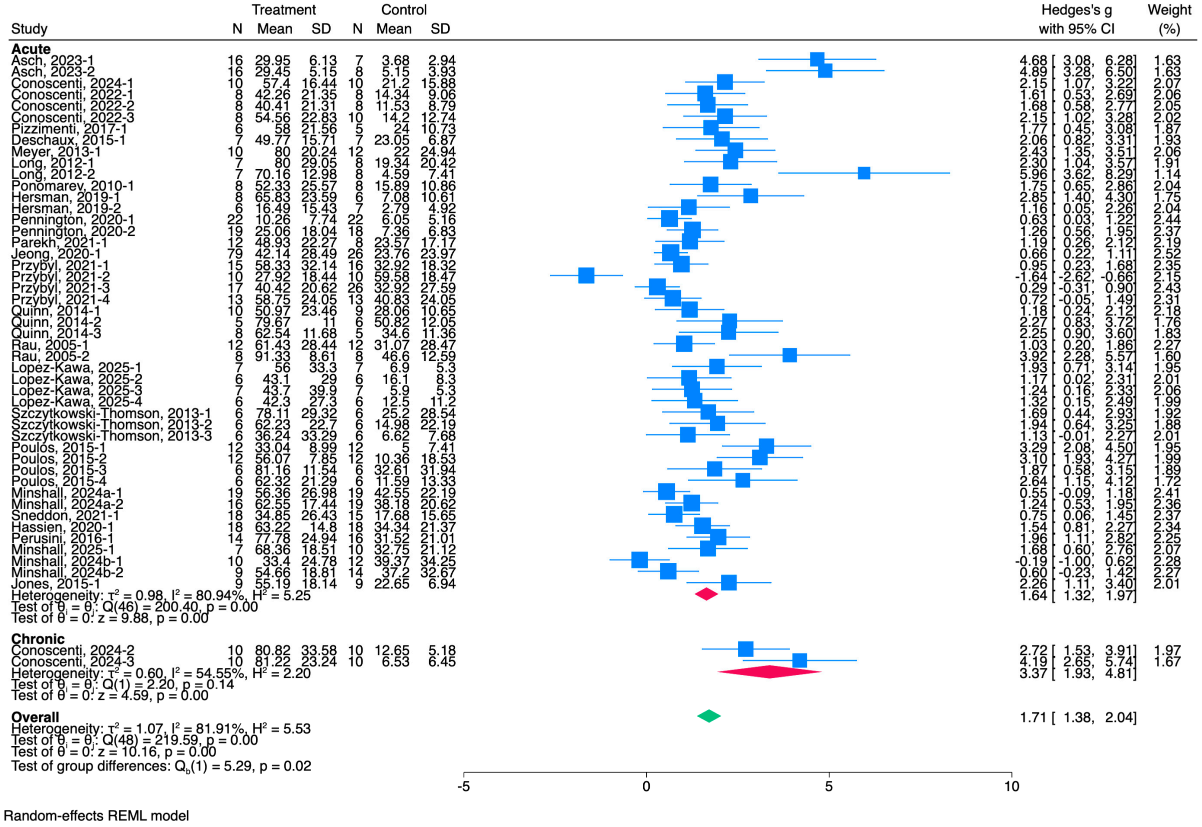

Figure S8. Subgroup forest plot by stress type.

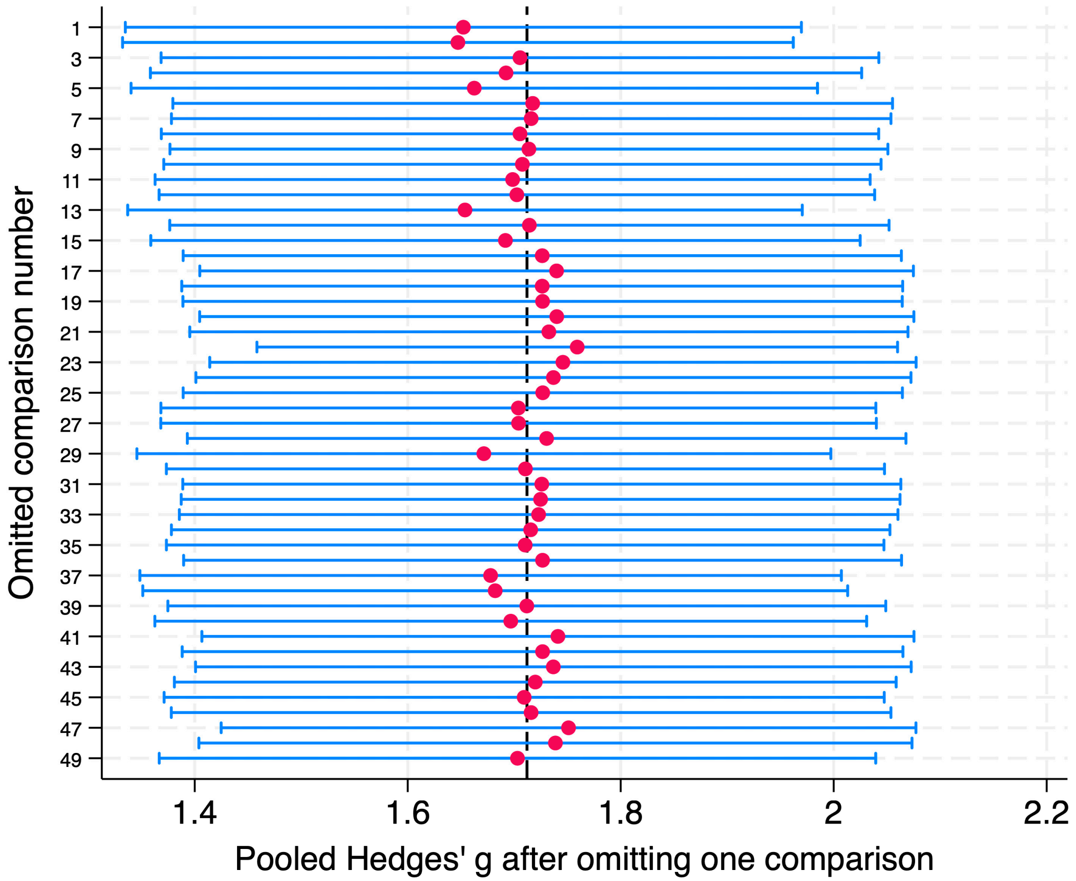

Figure S9. Leave-one-out sensitivity analysis. Each point represents the pooled Hedges' g after omitting one comparison. The vertical dashed line indicates the overall pooled effect from the main analysis.

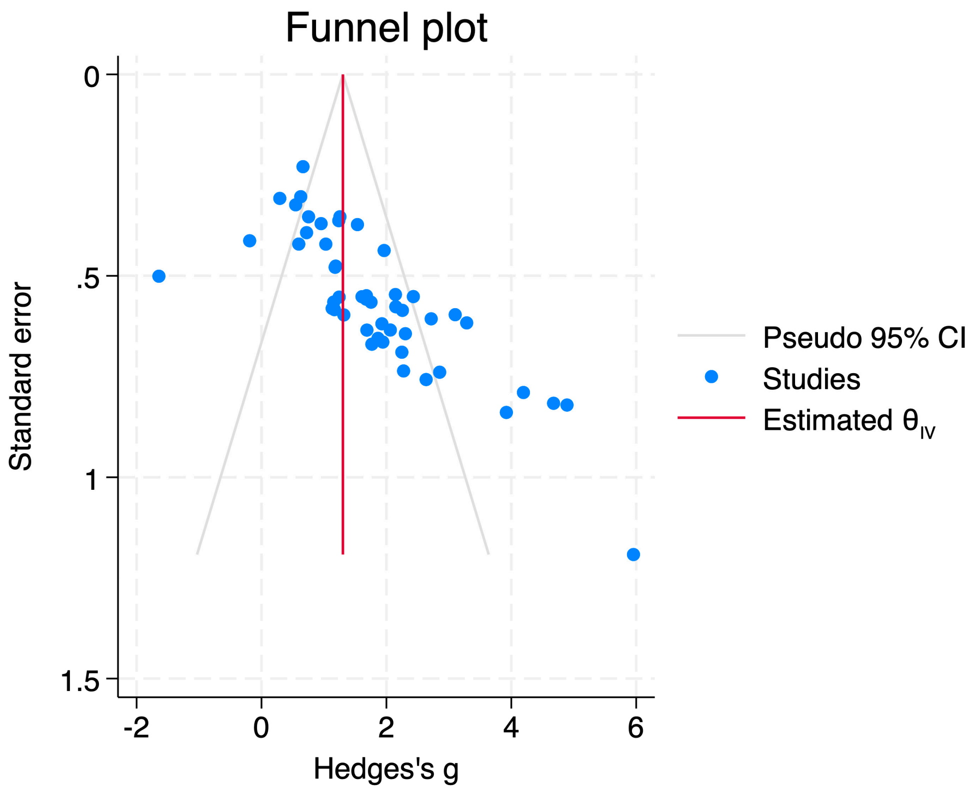

Figure S10. Funnel plot for the overall meta-analysis.

Study	Treatment N	Treatment Mean	Treatment SD	Control N	Control Mean	Control SD	Hedges's g with 95% CI	Weight (%)
Asch, 2023-1	16	29.95	6.13	7	3.68	2.94	4.68 [3.08, 6.28]	2.72
Conoscenti, 2024-1	10	57.4	16.44	10	21.2	15.88	2.15 [1.07, 3.22]	3.84
Conoscenti, 2022-1	8	42.26	21.35	8	14.34	9.06	1.61 [0.53, 2.69]	3.81
Pizzimenti, 2017-1	6	58	21.56	5	24	10.73	1.77 [0.45, 3.08]	3.29
Deschaux, 2015-1	7	49.77	15.71	7	23.05	6.87	2.06 [0.82, 3.31]	3.44
Meyer, 2013-1	10	80	20.24	12	22	24.94	2.43 [1.35, 3.51]	3.82
Long, 2012-1	7	80	29.05	8	19.34	20.42	2.30 [1.04, 3.57]	3.40
Ponomarev, 2010-1	8	52.33	25.57	8	15.89	10.86	1.75 [0.65, 2.86]	3.75
Hersman, 2019-1	8	65.83	23.59	6	7.08	10.61	2.85 [1.40, 4.30]	3.00
Pennington, 2020-1	22	10.26	7.74	22	6.05	5.16	0.63 [0.03, 1.22]	5.00
Parekh, 2021-1	12	48.93	22.27	8	23.57	17.17	1.19 [0.26, 2.12]	4.18
Jeong, 2020-1	79	42.14	28.49	26	23.76	23.97	0.66 [0.22, 1.11]	5.31
Przybyl, 2021-1	15	58.33	32.14	16	32.92	18.32	0.95 [0.23, 1.68]	4.69
Quinn, 2014-1	10	50.97	23.46	9	28.06	10.65	1.18 [0.24, 2.12]	4.16
Rau, 2005-1	12	61.43	28.44	12	31.07	28.47	1.03 [0.20, 1.86]	4.44
Lopez-Kawa, 2025-1	7	56	33.3	7	6.9	5.3	1.93 [0.71, 3.14]	3.51
Szczytkowski-Thomson, 2013-1	6	78.11	29.32	6	25.2	28.54	1.69 [0.44, 2.93]	3.44
Poulos, 2015-1	12	33.04	8.99	12	5	7.41	3.29 [2.08, 4.50]	3.52
Minshall, 2024a-1	19	56.36	26.98	19	42.55	22.19	0.55 [-0.09, 1.18]	4.91
Sneddon, 2021-1	18	34.85	26.43	15	17.68	15.65	0.75 [0.06, 1.45]	4.77
Hassien, 2020-1	18	63.22	14.8	18	34.34	21.37	1.54 [0.81, 2.27]	4.68
Perusini, 2016-1	14	77.78	24.94	16	31.52	21.01	1.96 [1.11, 2.82]	4.37
Minshall, 2025-1	7	68.36	18.51	10	32.75	21.12	1.68 [0.60, 2.76]	3.83
Minshall, 2024b-1	10	33.4	24.78	12	39.37	34.25	-0.19 [-1.00, 0.62]	4.49
Jones, 2015-1	9	55.19	18.14	9	22.65	6.94	2.26 [1.11, 3.40]	3.66
**Overall**							1.58 [1.22, 1.94]	

Heterogeneity: $\tau^2 = 0.59$, $I^2 = 74.23\%$, $H^2 = 3.88$

Test of $\theta_i = \theta_j$: Q(24) = 85.65, p = 0.00

Test of $\theta = 0$: z = 8.54, p = 0.00

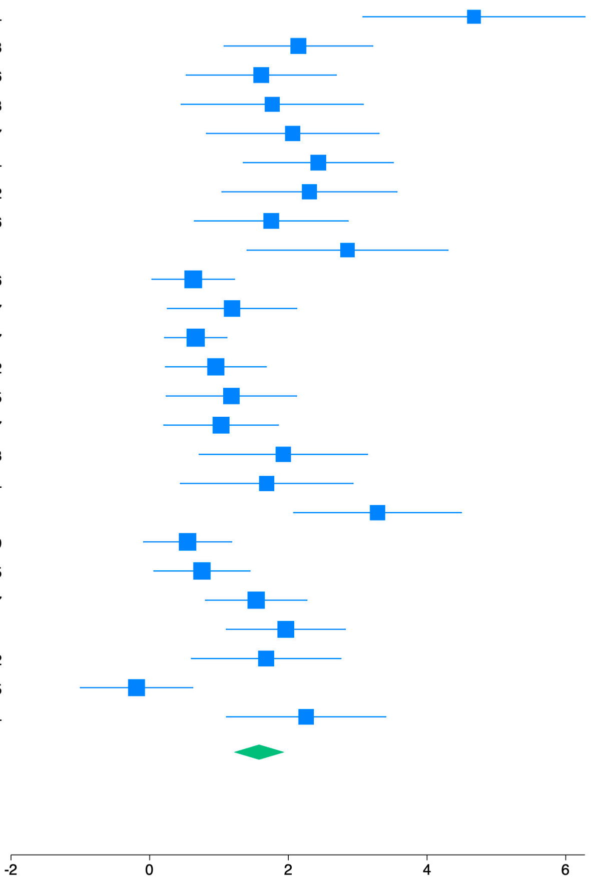

Random-effects REML model

Figure S11. Study-level sensitivity analysis including one representative comparison per study.

Study	Treatment N	Treatment Mean	Treatment SD	Control N	Control Mean	Control SD	Hedges's g with 95% CI	Weight (%)
Asch, 2023-1	16	29.95	6.13	7	3.68	2.94	4.68 [3.08, 6.28]	1.99
Asch, 2023-2	16	29.45	5.15	8	5.15	3.93	4.89 [3.28, 6.50]	1.98
Conoscenti, 2024-1	10	57.4	16.44	10	21.2	15.88	2.15 [1.07, 3.22]	2.60
Conoscenti, 2024-2	10	80.82	33.58	10	12.65	5.18	2.72 [1.53, 3.91]	2.46
Conoscenti, 2024-3	10	81.22	23.24	10	6.53	6.45	4.19 [2.65, 5.74]	2.04
Pizzimenti, 2017-1	6	58	21.56	5	24	10.73	1.77 [0.45, 3.08]	2.31
Deschaux, 2015-1	7	49.77	15.71	7	23.05	6.87	2.06 [0.82, 3.31]	2.39
Meyer, 2013-1	10	80	20.24	12	22	24.94	2.43 [1.35, 3.51]	2.59
Long, 2012-1	7	80	29.05	8	19.34	20.42	2.30 [1.04, 3.57]	2.37
Long, 2012-2	7	70.16	12.98	8	4.59	7.41	5.96 [3.62, 8.29]	1.34
Ponomarev, 2010-1	8	52.33	25.57	8	15.89	10.86	1.75 [0.65, 2.86]	2.55
Hersman, 2019-1	8	65.83	23.59	6	7.08	10.61	2.85 [1.40, 4.30]	2.15
Hersman, 2019-2	6	16.49	15.43	7	2.79	4.92	1.16 [0.05, 2.26]	2.55
Pennington, 2020-1	22	10.26	7.74	22	6.05	5.16	0.63 [0.03, 1.22]	3.14
Pennington, 2020-2	19	25.06	18.04	18	7.36	6.83	1.26 [0.56, 1.95]	3.04
Quinn, 2014-1	10	50.97	23.46	9	28.06	10.65	1.18 [0.24, 2.12]	2.76
Quinn, 2014-2	5	79.67	11	6	50.82	12.05	2.27 [0.83, 3.72]	2.16
Quinn, 2014-3	8	62.54	11.68	5	34.6	11.36	2.25 [0.90, 3.60]	2.26
Rau, 2005-1	12	61.43	28.44	12	31.07	28.47	1.03 [0.20, 1.86]	2.89
Rau, 2005-2	8	91.33	8.61	8	46.6	12.59	3.92 [2.28, 5.57]	1.94
Lopez-Kawa, 2025-1	7	56	33.3	7	6.9	5.3	1.93 [0.71, 3.14]	2.43
Lopez-Kawa, 2025-2	6	43.1	29	6	16.1	8.3	1.17 [0.02, 2.31]	2.51
Lopez-Kawa, 2025-3	7	43.7	39.9	7	5.9	5.3	1.24 [0.16, 2.33]	2.58
Lopez-Kawa, 2025-4	6	42.3	27.3	6	12.5	11.2	1.32 [0.15, 2.49]	2.48
Szczytkowski-Thomson, 2013-1	6	78.11	29.32	6	25.2	28.54	1.69 [0.44, 2.93]	2.39
Szczytkowski-Thomson, 2013-2	6	62.23	22.7	6	14.98	22.19	1.94 [0.64, 3.25]	2.32
Szczytkowski-Thomson, 2013-3	6	36.24	33.29	6	6.62	7.68	1.13 [-0.01, 2.27]	2.52
Poulos, 2015-1	12	33.04	8.99	12	5	7.41	3.29 [2.08, 4.50]	2.43
Poulos, 2015-2	12	56.07	7.85	12	10.36	18.53	3.10 [1.93, 4.27]	2.48
Poulos, 2015-3	6	81.16	11.54	6	32.61	31.94	1.87 [0.58, 3.15]	2.34
Poulos, 2015-4	6	62.32	21.29	6	11.59	13.33	2.64 [1.15, 4.12]	2.11
Minshall, 2024a-1	19	56.36	26.98	19	42.55	22.19	0.55 [-0.09, 1.18]	3.10
Minshall, 2024a-2	16	62.55	17.44	19	38.18	20.62	1.24 [0.53, 1.95]	3.02
Sneddon, 2021-1	18	34.85	26.43	15	17.68	15.65	0.75 [0.06, 1.45]	3.04
Hassien, 2020-1	18	63.22	14.8	18	34.34	21.37	1.54 [0.81, 2.27]	3.00
Perusini, 2016-1	14	77.78	24.94	16	31.52	21.01	1.96 [1.11, 2.82]	2.85
Minshall, 2025-1	7	68.36	18.51	10	32.75	21.12	1.68 [0.60, 2.76]	2.59
Minshall, 2024b-1	10	33.4	24.78	12	39.37	34.25	-0.19 [-1.00, 0.62]	2.91
Minshall, 2024b-2	9	54.66	18.81	14	37.2	32.67	0.60 [-0.23, 1.42]	2.89
Jones, 2015-1	9	55.19	18.14	9	22.65	6.94	2.26 [1.11, 3.40]	2.50
**Overall**							1.92 [1.57, 2.26]	

Heterogeneity: $\tau^2 = 0.90$, $I^2 = 76.68\%$, $H^2 = 4.29$

Test of $\theta_i = \theta_j$: Q(39) = 151.31, p = 0.00

Test of $\theta = 0$: z = 10.89, p = 0.00

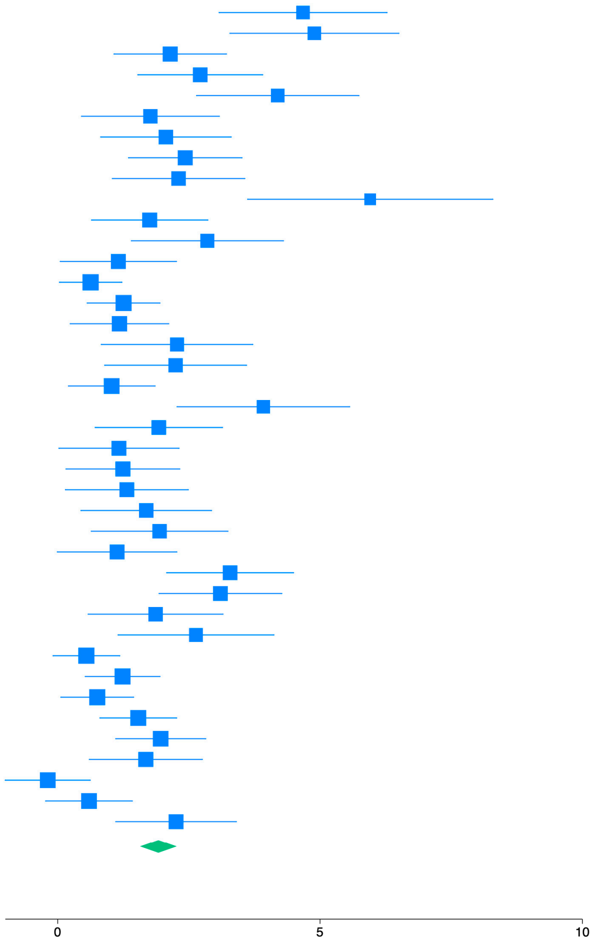

Random-effects REML model

Figure S12. Sensitivity analysis excluding non-footshock stress comparisons.
