# Supplementary material for: Stress-Enhanced Fear Learning in Rodents: A Systematic Review and Meta-Analysis of Fear-Learning Sensitization After Severe Stress
Source: Brain Sci. 2026 Jun 30;16(7):691. doi: 10.3390/brainsci16070691 (PMC13406739; doi:10.3390/brainsci16070691)
Supplement: Supplementary file 1 [file brainsci-16-00691-s001.zip › Supplementary_Methods_Search_Strategies.pdf]

Supplementary Methods. Search strategies

The complete original and expanded search strategies are listed below.

Search summary

Database	Original search	Expanded search	Records exported	Search date
PubMed	n=69	n=89	n=91	16 June 2026
Web of Science Core Collection	n=71	n=79	n=85	16 June 2026
Embase	n=104	n=113	n=123	18 June 2026
Scopus	n=77	n=93	n=98	18 June 2026

1. PubMed

Search date: 16 June 2026.
Limits: English language; publication date from database inception to 16 June 2026.

Original updated search: PTSD-related terms AND SEFL/fear-sensitization terms (n=69)

```
(
 "Stress Disorders, Post-Traumatic"[Mesh]
 OR "Post Traumatic Stress Disorder"[tiab]
 OR "Stress Disorder, Post Traumatic"[tiab]
 OR "Post-Traumatic Stress Disorder"[tiab]
 OR "Neuroses, Post-Traumatic"[tiab]
 OR "Neuroses, Post Traumatic"[tiab]
 OR "Post-Traumatic Neuroses"[tiab]
 OR "PTSD"[tiab]
 OR "Stress Disorder, Post-Traumatic"[tiab]
 OR "Post-Traumatic Stress Disorders"[tiab]
 OR "Post Traumatic Stress Disorders"[tiab]
 OR "Post-traumatic Stress Disorders"[tiab]
 OR "Post-traumatic Stress Disorder"[tiab]
 OR "Stress Disorder, Post-traumatic"[tiab]
 OR "Stress Disorders, Post traumatic"[tiab]
 OR "Neuroses, Post-traumatic"[tiab]
 OR "Post-traumatic Neuroses"[tiab]
 OR "Acute Post-Traumatic Stress Disorder"[tiab]
 OR "Acute Post Traumatic Stress Disorder"[tiab]
 OR "Chronic Post-Traumatic Stress Disorder"[tiab]
 OR "Chronic Post Traumatic Stress Disorder"[tiab]
 OR "Delayed Onset Post-Traumatic Stress Disorder"[tiab]
 OR "Delayed Onset Post Traumatic Stress Disorder"[tiab]
 OR "Moral Injury"[tiab]
 OR "Injury, Moral"[tiab]
 OR "Moral Injuries"[tiab]
)
AND
(
 "stress-enhanced fear learning"[tiab]
 OR "stress enhanced fear learning"[tiab]
 OR "SEFL"[tiab]
 OR "stress-induced enhancement of fear learning"[tiab]
 OR "stress induced enhancement of fear learning"[tiab]
 OR "stress-induced fear learning enhancement"[tiab]
 OR "fear learning sensitization"[tiab]
 OR "fear sensitization"[tiab]
 OR "stress-potentiated fear learning"[tiab]
 OR "stress potentiated fear learning"[tiab]
 OR "trauma-enhanced fear learning"[tiab]
 OR "trauma enhanced fear learning"[tiab]
 OR "stress-enhanced fear conditioning"[tiab]
 OR "stress enhanced fear conditioning"[tiab]
)
AND english[lang]
AND ("1800/01/01"[Date - Publication] : "2026/06/16"[Date - Publication])
```

Expanded SEFL-specific search not requiring PTSD-related terms (n=89)

```
(
  "stress-enhanced fear learning"[tiab]
  OR "stress enhanced fear learning"[tiab]
  OR "SEFL"[tiab]
  OR "trauma-enhanced fear learning"[tiab]
  OR "trauma enhanced fear learning"[tiab]
  OR "stress-enhanced fear conditioning"[tiab]
  OR "stress enhanced fear conditioning"[tiab]
  OR "stress-induced enhancement of fear learning"[tiab]
  OR "stress induced enhancement of fear learning"[tiab]
  OR "stress-induced fear learning enhancement"[tiab]
  OR "fear learning sensitization"[tiab]
  OR "fear sensitization"[tiab]
  OR "stress-potentiated fear learning"[tiab]
  OR "stress potentiated fear learning"[tiab]
)
AND
(
  rat[tiab]
  OR rats[tiab]
  OR mouse[tiab]
  OR mice[tiab]
  OR rodent[tiab]
  OR rodents[tiab]
  OR "Rats"[Mesh]
  OR "Mice"[Mesh]
)
AND english[lang]
AND ("1800/01/01"[Date - Publication] : "2026/06/16"[Date - Publication])
```

Records exported from PubMed: n=91.

2. Web of Science Core Collection

Search date: 16 June 2026.
Limits: English language; publication date from database inception to 16 June 2026.

Original updated search: PTSD-related terms AND SEFL/fear-sensitization terms (n=71)

Topic:
"post-traumatic stress disorder" OR "post traumatic stress disorder" OR "posttraumatic stress disorder" OR "post-traumatic stress disorders" OR "post traumatic stress disorders" OR "posttraumatic stress disorders" OR PTSD OR "moral injury" OR "moral injuries"
AND Topic:
"stress-enhanced fear learning" OR "stress enhanced fear learning" OR SEFL OR "stress-induced enhancement of fear learning" OR "stress induced enhancement of fear learning" OR "stress-induced fear learning enhancement" OR "fear learning sensitization" OR "fear sensitization" OR "stress-potentiated fear learning" OR "stress potentiated fear learning" OR "trauma-enhanced fear learning" OR "trauma enhanced fear learning" OR "stress-enhanced fear conditioning" OR "stress enhanced fear conditioning"

Expanded SEFL-specific search: SEFL/fear-sensitization terms AND rodent terms (n=79)

Topic:
"stress-enhanced fear learning" OR "stress enhanced fear learning" OR SEFL OR "stress-induced enhancement of fear learning" OR "stress induced enhancement of fear learning" OR "stress-induced fear learning enhancement" OR "fear learning sensitization" OR "fear sensitization" OR "stress-potentiated fear learning" OR "stress potentiated fear learning" OR "trauma-enhanced fear learning" OR "trauma enhanced fear learning" OR "stress-enhanced fear conditioning" OR "stress enhanced fear conditioning"
AND Topic:
rat OR rats OR mouse OR mice OR rodent OR rodents

Records exported from Web of Science Core Collection: n=85.

3. Embase

Search date: 18 June 2026.
Limits: English language; publication date from database inception to 18 June 2026.

Original updated search: PTSD-related terms AND SEFL/fear-sensitization terms (n=104)

('post-traumatic stress disorder',ab OR 'stress disorder, post-traumatic',ab OR 'post traumatic stress disorder',ab OR 'neuroses, post-traumatic',ab OR 'neuroses, post traumatic',ab OR 'post-traumatic neuroses',ab OR 'ptsd',ab OR 'stress disorder, post traumatic',ab OR 'post-traumatic stress disorders',ab OR 'post traumatic stress disorders',ab OR 'posttraumatic stress disorders',ab OR 'posttraumatic stress disorder',ab OR 'stress disorder, posttraumatic',ab OR 'stress disorders, posttraumatic',ab OR 'neuroses, posttraumatic',ab OR 'posttraumatic neuroses',ab OR 'acute post-traumatic stress disorder',ab OR 'acute post traumatic stress disorder',ab OR 'chronic post-traumatic stress disorder',ab OR 'chronic post traumatic stress disorder',ab OR 'delayed onset post-traumatic stress disorder',ab OR 'delayed onset post traumatic stress disorder',ab OR 'moral injury',ab OR 'injury, moral',ab OR 'moral injuries',ab)
AND
('stress-enhanced fear learning',ab OR 'stress enhanced fear learning',ab OR 'sefl',ab OR 'stress-induced enhancement of fear learning',ab OR 'stress induced enhancement of fear learning',ab OR 'stress-induced fear learning enhancement',ab OR 'fear learning sensitization',ab OR 'fear sensitization',ab OR 'stress-potentiated fear learning',ab OR 'stress potentiated fear learning',ab OR 'trauma-enhanced fear learning',ab OR 'trauma enhanced fear learning',ab OR 'stress-enhanced fear conditioning',ab OR 'stress enhanced fear conditioning',ab)

Expanded SEFL-specific search without the PTSD block (n=113)

('stress-enhanced fear learning',ab OR 'stress enhanced fear learning',ab OR 'sefl',ab OR 'stress-induced enhancement of fear learning',ab OR 'stress induced enhancement of fear learning',ab OR 'stress-induced fear learning enhancement',ab OR 'fear learning sensitization',ab OR 'fear sensitization',ab OR 'stress-potentiated fear learning',ab OR 'stress potentiated fear learning',ab OR 'trauma-enhanced fear learning',ab OR 'trauma enhanced fear learning',ab OR 'stress-enhanced fear conditioning',ab OR 'stress enhanced fear conditioning',ab OR 'stress-enhanced contextual fear conditioning',ab OR 'stress enhanced contextual fear conditioning',ab OR 'trauma-enhanced contextual fear conditioning',ab OR 'trauma enhanced contextual fear conditioning',ab OR 'prior stress enhanced fear learning',ab OR 'prior stress-enhanced fear learning',ab)

Records exported from Embase: n=123.

4. Scopus

Search date: 18 June 2026.
Limits: English language; publication date from database inception to 18 June 2026.

Original updated search: PTSD-related terms AND SEFL/fear-sensitization terms (n=77)

TITLE-ABS-KEY ("Stress Disorders, Post-Traumatic" OR "Post-Traumatic Stress Disorder" OR "Stress Disorder, Post-Traumatic" OR "Post Traumatic Stress Disorder" OR "Neuroses, Post-Traumatic" OR "Neuroses, Post Traumatic" OR "Post-Traumatic Neuroses" OR "PTSD" OR "Stress Disorder, Post Traumatic" OR "Post-Traumatic Stress Disorders" OR "Post Traumatic Stress Disorders" OR "Posttraumatic Stress Disorders" OR "Posttraumatic Stress Disorder" OR "Stress Disorder, Posttraumatic" OR "Stress Disorders, Posttraumatic" OR "Neuroses,

Posttraumatic" OR "Posttraumatic Neuroses" OR "Acute Post-Traumatic Stress Disorder" OR "Acute Post Traumatic Stress Disorder" OR "Chronic Post-Traumatic Stress Disorder" OR "Chronic Post Traumatic Stress Disorder" OR "Delayed Onset Post-Traumatic Stress Disorder" OR "Delayed Onset Post Traumatic Stress Disorder" OR "Moral Injury" OR "Injury, Moral" OR "Moral Injuries")
AND
TITLE-ABS-KEY ("stress-enhanced fear learning" OR "stress enhanced fear learning" OR "SEFL" OR "stress-induced enhancement of fear learning" OR "stress induced enhancement of fear learning" OR "stress-induced fear learning enhancement" OR "fear learning sensitization" OR "fear sensitization" OR "stress-potentiated fear learning" OR "stress potentiated fear learning" OR "trauma-enhanced fear learning" OR "trauma enhanced fear learning" OR "stress-enhanced fear conditioning" OR "stress enhanced fear conditioning")

Expanded SEFL-specific search without the PTSD block (n=93)

TITLE-ABS-KEY ("stress-enhanced fear learning" OR "stress enhanced fear learning" OR "SEFL" OR "stress-induced enhancement of fear learning" OR "stress induced enhancement of fear learning" OR "stress-induced fear learning enhancement" OR "fear learning sensitization" OR "fear sensitization" OR "stress-potentiated fear learning" OR "stress potentiated fear learning" OR "trauma-enhanced fear learning" OR "trauma enhanced fear learning" OR "stress-enhanced fear conditioning" OR "stress enhanced fear conditioning" OR "stress-enhanced contextual fear conditioning" OR "stress enhanced contextual fear conditioning" OR "trauma-enhanced contextual fear conditioning" OR "trauma enhanced contextual fear conditioning" OR "prior stress enhanced fear learning" OR "prior stress-enhanced fear learning")

Records exported from Scopus: n=98.
